# East‒West genetic differentiation across the Indo-Burma hotspot: evidence from two closely related dioecious figs

**DOI:** 10.1186/s12870-023-04324-6

**Published:** 2023-06-16

**Authors:** Jian-Feng Huang, Shu-Qiong Li, Rui Xu, Yan-Qiong Peng

**Affiliations:** 1 CAS Key Laboratory of Tropical Forest Ecoloy, Xishuangbanna Tropical Botanical Garden, Chinese Academy of Sciences, Mengla, 666303 China; 2 University of Chinese Academy of Sciences, Beijing, 100049 China; 3 Yunnan Academy of Biodiversity/College of Biodiversity and Conservation, Southwest Forestry University, Kunming, China

**Keywords:** Indo-Burma, Phylogeography, East‒West differentiation, *Ficus*, Pollinating wasp

## Abstract

**Background:**

Understanding biodiversity patterns and their underlying mechanisms is of interest to ecologists, biogeographers and conservationists and is critically important for conservation efforts. The Indo-Burma hotspot features high species diversity and endemism, yet it also faces significant threats and biodiversity losses; however, few studies have explored the genetic structure and underlying mechanisms of Indo-Burmese species. Here, we conducted a comparative phylogeographic analysis of two closely related dioecious *Ficus* species, *F. hispida* and *F. heterostyla*, based on wide and intensive population sampling across Indo-Burma ranges, using chloroplast (psbA-trnH, trnS-trnG) and nuclear microsatellite (nSSR) markers, as well as ecological niche modeling.

**Results:**

The results indicated large numbers of population-specific cpDNA haplotypes and nSSR alleles in the two species. *F. hispida* showed slightly higher chloroplast diversity but lower nuclear diversity than *F. heterostyla*. Low-altitude mountainous areas of northern Indo-Burma were revealed to have high genetic diversity and high habitat suitability, suggesting potential climate refugia and conservation priority areas. Strong phylogeographic structure and a marked east‒west differentiation pattern were observed in both species, due to the interactions between biotic and abiotic factors. Interspecific dissimilarities at fine-scale genetic structure and asynchronized historical dynamics of east‒west differentiation between species were also detected, which were attributed to different species-specific traits.

**Conclusions:**

We confirm hypothesized predictions that interactions between biotic and abiotic factors largely determine the patterns of genetic diversity and phylogeographic structure of Indo-Burmese plants. The east‒west genetic differentiation pattern observed in two targeted figs can be generalized to some other Indo-Burmese plants. The results and findings of this work will contribute to the conservation of Indo-Burmese biodiversity and facilitate targeted conservation efforts for different species.

**Supplementary Information:**

The online version contains supplementary material available at 10.1186/s12870-023-04324-6.

## Background

Patterns of biodiversity and their underlying mechanisms are among the central issues in ecology and biogeography and provide core knowledge for conservation [1–4]. The distributions and patterns of genetic diversity and structure of plants and animals are largely determined by a number of abiotic (e.g., climactic oscillations, geologic processes) and biotic factors (e.g., climate sensitivity, pollen and propagule dispersal, life history traits) [5–12]. In particular, the climatic oscillations in the Quaternary are well known to have profoundly affected the geographical distribution of much of Earth’s biota [6, 13]. Comparative phylogeography of multiple species has proven powerful in revealing common and idiosyncratic patterns among codistributed organisms as well as the abiotic and biotic causes [14–21].

Indo-Burma, covering Burma, Thailand, Laos, Cambodia, Vietnam and parts of southern China, northeast India and Bangladesh, is a global hotspot of biodiversity in both plants and animals. The 2,373,000 km^2^ Indo-Burma hotspot supports ca. 13,500 plant species, of which approximately 7,000 were estimated to be endemic to this hotspot [22, 23]. Complex geological and evolutionary histories, as well as highly diverse habitats mainly due to the wide variation in landform, climate, and latitude, were deemed to support the high species diversity. The isolated habitats caused by periods of high sea level and vegetation changes during the glacial episodes of the Pleistocene may have largely contributed to the high endemism [1, 23, 24]. Meanwhile, Indo-Burma is also considered one of the world’s most threatened terrestrial eco-regions by factors such as human population growth, deforestation and habitat conversion, resource exploitation, pollution and global warming [25, 26].

Along with the improvement of the political environment and infrastructure in regions of Indo-Burma in recent years, it has become more feasible for researchers to enter large parts of this hotspot and explore biodiversity [27]. For example, a few phylogeographic case studies have been conducted among Indo-Burmese fauna [e.g., 28‒37]. The population genetic investigation of the endemic species *Dalbergia cochinchinensis* and *D. oliveri* represents the first detailed analysis of landscape genetics for tree species within Thailand and revealed that drainage has shaped their phylogeographic structures [27]. Nevertheless, most of these studies were local and did not cover the entire Indo-Burma region. Our recent study on monoecious *F. altissima* is the first to investigate phylogeographic patterns of plants based on samples collected widely across Indo-Burma. A homogenized phylogeographic structure within the Indo-Burma hotspot was revealed, mainly due to extensive wasp-mediated pollen flow [38]. Nevertheless, our knowledge about the genetic diversity and pattern, as well as the underlying mechanisms among Indo-Burmese species, is extremely poor, especially for plants. These underresearched conditions also impede biodiversity conservation programs in this region.

*Ficus* (Moraceae) is a pantropical and hyperdiverse genus (ca. 850 species) with a large range of growth forms [39, 40]. Members of this genus are the center of an intricate web of specialist and generalist animals and are considered keystone plant resources in many tropical ecosystems [41]. They are well known for their specialized inflorescence (syconium or fig) and their intricate relationships with their species-specific pollinating fig wasps (Hymenoptera: Agaonidae) [42–44]. Monoecious species produce wasps and seeds in the same fig, while dioecious species produce wasps and seeds in separate male and female figs on different trees [45]. Monoecious and dioecious figs appear to be associated with divergent suites of characters (e.g., life form, population density, fruiting frequency, pollinator dispersal ecology). For example, monoecious figs are often tall trees that reach the canopy and are associated with lower population density and lower endemism. In contrast, dioecious species are usually understory small trees or shrubs, with high local population density and high endemism [46, 47]. Pollinators of monoecious figs seem to disperse much farther than the pollinators of dioecious figs [38, 47–49]. Therefore, dioecious fig trees are often more genetically structured than monoecious fig trees [50–54] and are expected to be a better study system for disclosing the phylogeographic patterns of species in response to climatic oscillations and geological events.

In this study, we focused on the spatial genetic diversity and structure of two closely related dioecious *Ficus* species, *F. hispida* and *F. heterostyla*, based on wide and intensive population sampling across Indo-Burma ranges and evidence from chloroplast and nuclear markers, as well as ecological niche modeling. These two plants are different in fruiting position (Fig. S1), which affects the spread of seed and pollen; thus may produce species-specific phylogeographic patterns at fine-scale. Here, we aimed to (1) investigate the spatial distribution of genetic diversity across Indo-Burma; (2) reveal the interspecific similarities and dissimilarities in phylogeographical structure; and (3) explore the underlying biotic and abiotic mechanisms that influenced the phylogeographical structure of the two focal figs.

## Results

### Genetic diversity

The genetic diversity parameters for the studied populations are summarized in Table 1. We generated both cpDNA intergenic spacers for 326 *F. hispida* individuals and 276 *F. heterostyla* individuals. The combined data of the two spacers resulted in an alignment of 1058 bp. In total, 50 haplotypes were detected with 19 single nucleotide polymorphisms and 25 indels, representing 35 and 24 haplotypes for *F. hispida* and *F. heterostyla*, respectively. Nine haplotypes were shared by both species. Within species, 26 of the 35 (74.3%) *F. hispida* haplotypes and 19 of the 24 (79.2%) *F. heterostyla* haplotypes exclusively occurred in one population. Meanwhile, 14 of the 30 (46.7%) *F. hispida* populations and 13 of 21 (61.9%) the *F. heterostyla* populations were revealed to have unique haplotypes. Seventeen populations of *F. hispida* had only a single haplotype, while the remaining thirteen populations had haplotype diversity *H*_d_ values ranging from 0.100 to 1.000 and nucleotide diversity π (× 10^–2^) values ranging from 0.054 to 0.862. For *F. heterostyla*, twelve populations had only a single haplotype, while the remaining nine populations had *H*_d_ values between 0.143 and 0.667 and π (× 10^–2^) values between 0.072 and 0.253. A pattern of high haplotype diversity and low nucleotide diversity at the species level was revealed in both figs. Both haplotype and nucleotide diversities were higher in *F. hispida* (*H*_d_ = 0.934, π = 0.00504) than in *F. heterostyla* (*H*_d_ = 0.922, π = 0.00391) (Table 1).

**Table 1:** Sampling informations and parameters of genetic diversity of *F. hispida* and *F. heterostyla*, averaged across two cpDNA regions and 14 nuclear microsatellite loci

Species	Code	Sample site	Lat.(N)	Long.(E)	Alt.(m)	n	cpDNA				nuclear SSR			
							*H*	*H*_d_	π (10^− 2)^		*N*_a_	*PA*	*H*_O_	*H*_E_
***F. hispida***	D-cyd	Yunnan, China	24.411	98.565	903	8	H1, H2	0.536	0.105		4.21	0.57	0.547	0.519
	D-cyl	Yunnan, China	26.08	98.848	810	4	H1	0	0		2.57	0	0.524	0.417
	D-cyx	Yunnan, China	22.129	100.667	695	20	H1, **H3**	0.100	0.078		4.79	0.21	0.58	0.524
	D-cgp	Guangxi, China	22.15	106.782	340	4	H4, **H5**, **H6**, **H7**	1	0.862		3.14	0.43	0.500	0.540
	D-cgb	Guangxi, Chia	21.76	107.405	262	5	**H8**	0	0		3.21	0.21	0.557	0.473
	D-cgn	Guangxi, China	23.331	107.964	157	7	H4	0	0		3.93	0.07	0.551	0.515
	D-chc	Hainan, China	19.105	109.104	201	6	H9	0	0		2.71	0	0.429	0.413
	D-chd	Hainan, China	18.666	109.914	550	6	H10	0	0		3.21	0	0.464	0.426
	D-cgl	Guangdong, China	20.949	110.1	5	5	H9	0	0		2.79	0	0.443	0.417
	D-cgg	Guangdong, China	21.925	111.841	270	15	H4, H9	0.419	0.289		4.00	0.36	0.454	0.469
	D-mms	Mandalay, Myanmar	22.549	95.998	662	14	H1	0	0		3.64	0.14	0.449	0.435
	D-msk	Shan, Myanmar	20.698	96.507	653	12	H2, **H11**	0.167	0.182		4.86	0.21	0.565	0.563
	D-mbt	Bago, Myanmar	18.981	96.514	467	19	H2, **H12**, H13, **H14**	0.626	0.144		5.21	0.29	0.545	0.538
	D-myh	Yangon, Myanmar	17.304	96.165	16	16	H1	0	0		4.50	0	0.565	0.563
	D-mmm	Moulmein, Myanmar	16.419	97.673	214	14	H1, H13, H15	0.59	0.292		4.14	0	0.418	0.418
	D-mmy	Mon, Myanmar	15.139	97.828	10	19	H13	0	0		4.14	0.14	0.466	0.450
	D-tra	Ranong, Thailand	10.511	98.908	66	9	**H16**	0	0		2.93	0.07	0.421	0.378
	D-ttn	Trang, Thailand	7.544	99.793	136	11	**H17**, **H18**, **H19**, **H20**	0.75	0.351		3.50	0	0.374	0.381
	D-tpl	Phetchabun, Thailand	16.743	101.3	205	8	**H21**	0	0		3.07	0.07	0.411	0.439
	D-tch	Chantaburi, Thailand	12.674	102.096	76	6	**H22**	0	0		3.77	0.29	0.405	0.492
	D-tmn	Mukdahan, Thailand	16.41	104.428	184	14	H23	0	0		4.50	0.21	0.495	0.537
	D-llo	Louangphrabang, Laos	19.851	102.165	419	19	H15, **H24**, **H25**, **H26**, **H27**, **H28**	0.655	0.272		5.27	0.21	0.474	0.515
	D-lvx	Vientiane, Laos	18.071	102.674	316	20	H15, **H29**, **H30**	0.468	0.262		4.64	0.21	0.454	0.501
	D-ckk	Kampot, Cambodia	10.583	104.083	204	10	H10	0	0		3.14	0	0.548	0.429
	D-vhn	Ha Tinh, Vietnam	18.648	105.718	na	4	**H31**, **H32**	0.5	0.198		2.64	0	0.446	0.384
	D-vks	Kon Tum, Vietnam	14.394	107.825	827	4	**H33**	0	0		2.93	0.07	0.607	0.487
	D-vtp	Hue, Vietnam	16.22	108.1	na	11	H10, H23	0.182	0.054		4.07	0.07	0.487	0.522
	D-vdl	Dark Lak, Vietnam	12.415	108.177	493	14	H10	0	0		3.86	0	0.408	0.378
	D-vkh	Khanh Hoa, Vietnam	12.159	109.043	25	11	H34	0	0		3.93	0.21	0.468	0.464
	D-vpy	Phu Yen, Vietnam	12.852	109.391	28	11	H10, H34, **H35**	0.511	0.245		3.50	0	0.448	0.474
	mean										3.76	0.14	0.483	0.469
	species							0.934	0.504		14.43	na	0.481	0.573
*** F. heterostyla***	H-cyx	Yunnan, China	21.907	101.28	595	15	H1, **H36**	0.419	0.245		9.07	0.57	0.653	0.790
	H-cgp	Guangxi, China	22.042	106.767	437	10	**H7**	0	0		6.29	0.14	0.745	0.742
	H-cgb	Guangxi, China	21.765	107.293	562	13	**H37**	0	0		8.57	0.14	0.769	0.796
	H-mmk	Kyaikto, Myanmar	17.274	97.198	58	12	H1, **H38**, **H39**	0.667	0.102		7.36	0.21	0.680	0.742
	H-tka	Karnchanaburi, Thailand	14.98	98.63	253	13	H22	0	0		6.21	0.21	0.655	0.667
	H-tcm	Chiengmai, Thailand	18.894	98.858	673	10	H1, **H40**, **H41**, **H42**, **H43**	0.667	0.213		7.00	0.71	0.750	0.746
	H-tra	Ranong, Thailand	10.511	98.908	66	3	H22, H33	0.667	0.132		1.94	0.07	0.810	0.429
	H-tcd	Chiengmai, Thailand	18.81	98.914	1090	12	H1, **H44**	0.200	0.078		7.21	0.50	0.732	0.734
	H-tta	Tak, Thailand	16.791	98.919	810	14	H1, **H13**	0.385	0.113		9.07	0.29	0.667	0.812
	H-tsb	Suphan Buri, Thailand	14.98	99.322	585	15	H15	0	0		6.93	0.64	0.586	0.618
	H-tla	Lampang, Thailand	18.841	99.467	540	13	H15	0	0		10.36	0.21	0.846	0.806
	H-tko	Korat, Thailand	14.482	101.384	512	14	**H45**, **H46**	0.143	0.072		5.24	0.14	0.469	0.550
	H-tch	Chanthaburi, Thailand	12.674	102.096	76	17	H22	0	0		5.07	0.14	0.496	0.481
	H-tpb	Prachin Buri, Thailand	13.994	102.206	162	16	**H47**	0	0		4.71	0.14	0.512	0.456
	H-tur	Ubon Ratchathani, Thailand	14.446	105.215	179	12	**H9**	0	0		4.57	0	0.524	0.567
	H-lpn	Xieng Khuang, Laos	20.363	102.39	328	12	H15, **H48**, **H49**	0.591	0.113		8.00	0.21	0.690	0.783
	H-cbh	Kampot, Cambodia	10.811	104.279	204	16	H10, **H50**	0.513	0.253		4.50	0.14	0.460	0.476
	H-vcm	Kon Tum, Vietnam	14.5	107.742	722	20	H33	0	0		5.43	0	0.538	0.612
	H-vdl	Dac Lac, Vietnam	12.415	108.177	493	11	H22	0	0		4.07	0.07	0.493	0.500
	H-vkh	Khanh Hoa, Vietnam	12.121	109.001	25	11	**H34**	0	0		3.93	0	0.584	0.530
	H-vpy	Phu Yen, Vietnam	12.852	109.391	28	17	H10	0	0		4.64	0	0.604	0.576
	mean										6.20	0.22	0.632	0.639
	species							0.922	0.391		23.86	na	0.617	0.865

Note: na, no available data. The bold indicated the unique haplotype at species level

Microsatellite genotype data were obtained for 315 *F. hispida* and 256 *F. heterostyla* individuals. Across 14 loci, 202 alleles were identified in 30 *F. hispida* populations, corresponding to 14.43 alleles per locus and ranging from 5 to 22 alleles for individual loci, while 334 alleles were identified in 21 *F. heterostyla* populations, corresponding to 23.86 alleles per locus and ranging from 12 to 34 alleles for individual loci. A species-level *H*_O_ of 0.481 and an *H*_E_ of 0.573 were observed for *F. hispida*, and an *H*_O_ of 0.632 and an *H*_E_ of 0.639 were observed for *F. heterostyla*. At the population level, *N*_a_ ranged from 2.57 to 5.29 (average 3.76), *PA* ranged from 0.00 to 0.57 (average 0.14), *H*_O_ ranged from 0.374 to 0.607 (average 0.483) and *H*_E_ ranged from 0.378 to 0.563 (average 0.469) among *F. hispida* populations, while *N*_a_ ranged from 1.92 to 10.36 (average 6.20), *PA* ranged from 0.00 to 0.71 (average 0.22), *H*_O_ ranged from 0.460 to 0.846 (average 0.632) and *H*_E_ ranged from 0.429 to 0.812 (average 0.639) among *F. heterostyla* populations. *F. heterostyla* showed a higher level of nuclear genetic diversity than *F. hispida* at both the species and population levels.

The values of *H*, *H*_d_, *N*_a_, *PA*, *H*_O_ and *H*_E_ are geographically displayed in Fig. S2, largely supporting a higher level of genetic diversity of both fig plants distributed in the northern part of the Indo-Burma hotspot.

### Phylogeographic structure

**Fig. 1:**
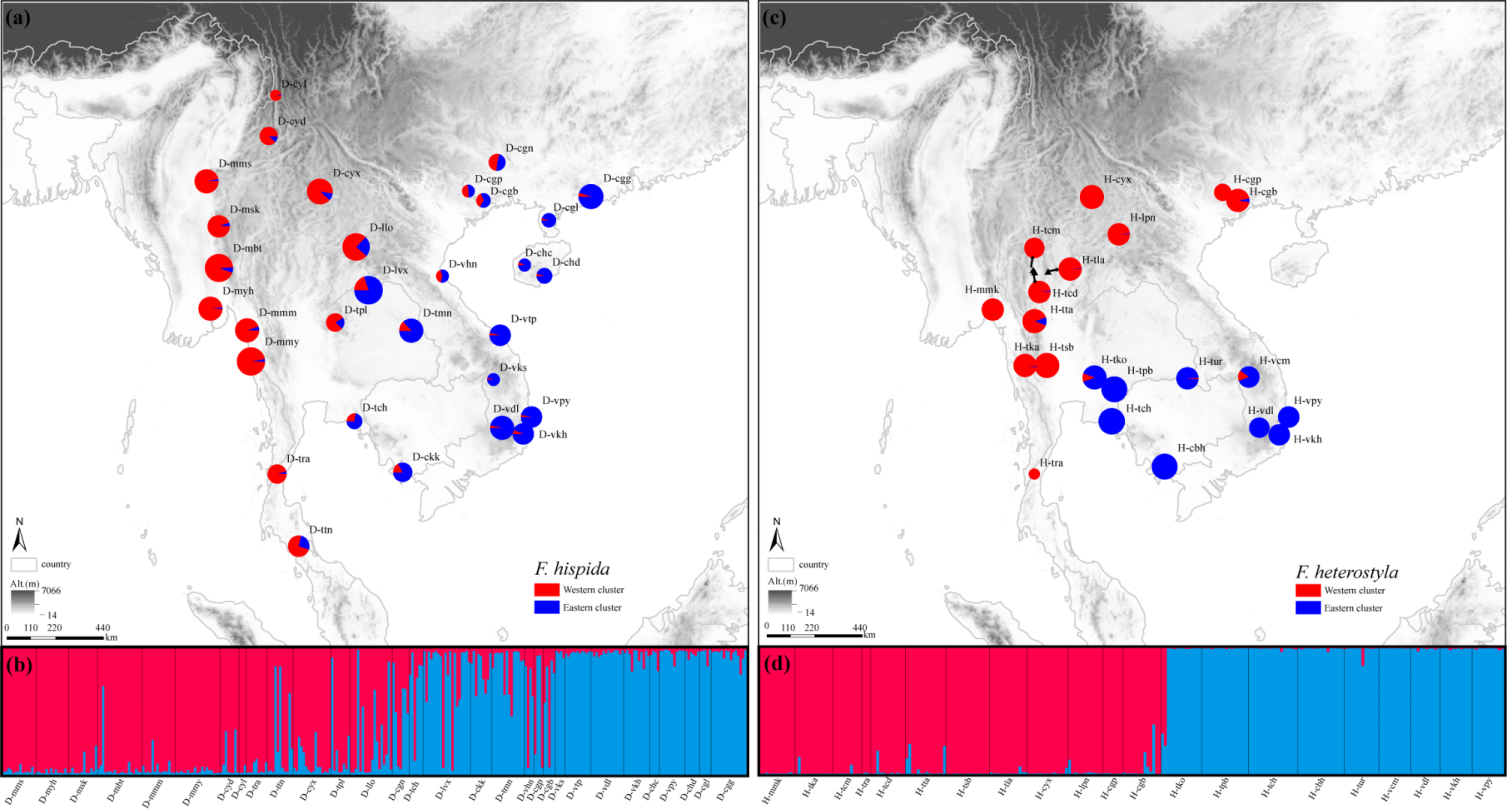
Geographical distribution of the genetic clusters detected by STRUCTURE for *F. hispida* (**a**) and *F. heterostyla* (**c**) and bar plots of the membership probabilities of *F. hispida* (**b**) and *F. heterostyla* (**d**) individuals to the different clusters from the STRUCTURE analysis at *K* = 2. The pie charts represent the assignment values of the admixed clustering analysis in (a) and (c). Solid black lines define the boundaries between populations in (b) and (d). The populations are roughly arranged according to longitude from west to east. Western clusters are colored red, and eastern clusters are colored blue

STRUCTURE HARVESTER analysis indicated an optimal *K* value of 2 using the delta*K* criterion for both target species. Two intraspecific clusters were weakly (*F. hispida*, Fig. 1b) or strongly (*F. heterostyla*, Fig. 1d) differentiated, which was roughly associated with geographical trends in longitude. Each population was assigned to a cluster if it had at least 50% membership within that cluster. Different from *F. heterostyla* populations showing relatively pure genetics, geographically close *F. hispida* populations from the different clusters presented genetic admixture, suggesting more intercluster pollen flow. The distribution of assignments showed east‒west geographic genetic structure of *F. hispida* and *F. heterostyla* (Fig. 1). The bar plots of the membership probabilities at *K* = 3 to 8 are also shown. A subcluster within the western *F. hispida* cluster including six westernmost populations (D-mms, D-myh, D-msk, D-mbt, D-cyd and D-cyl) (Fig. S3). Two subclusters within the eastern *F. heterostyla* cluster were obviously separated, one including populations H-tur, H-vcm, H-vdl, H-vkh, and H-vpy and another including populations H-tko, H-tpb, H-tch, and H-cbh (Fig. S4). A striking contrasting pattern between the two figs was revealed: *F. hispida* often showed heterogeneous genetic mixture among individuals of a population, while a high level of genetic homogeneity was observed within each population, subcluster or cluster of *F. heterostyla* (Fig. 1, S3 and S4).

**Fig. 2:**
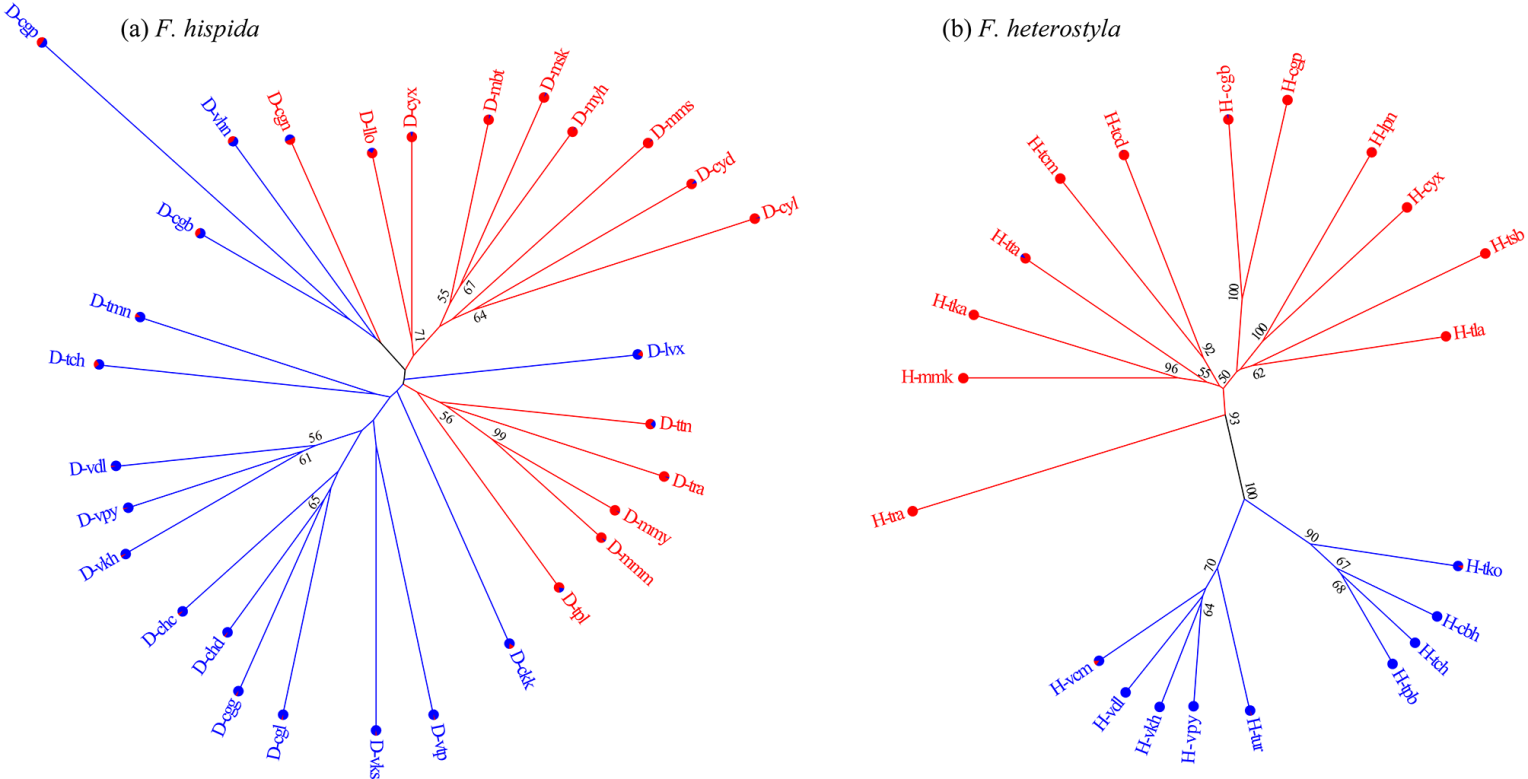
Unrooted neighbor-joining trees showing the relationships among 30 *F. hispida* (**a**) and 21 *F. heterostyla* populations (**b**) based on the chord distance (*Dc*) of Cavalli-Sforza and Edwards estimated from 14 nSSR loci. Bootstrap values (> 50%) calculated with 1000 replicates are given at the nodes. The trees are colored according to the results of the structure analysis, and the pie charts at the tips represent the assignment values of the admixed clustering analysis

**Fig. 3:**
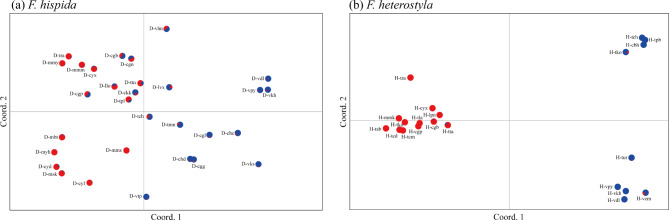
Two-dimensional scatter diagram based on principal coordinate analysis of genetic variation in the studied *F. hispida* (**a**) and *F. heterostyla* (**b**) populations. The pie charts represent the assignment values of the admixed clustering analysis

The neighbor-joining tree (Fig. 2) and PCoA (Fig. 3) revealed a pattern similar to that from STRUCTURE analysis. The differentiation between western and eastern populations, as well as the emerging subclusters, was supported by both neighbor-joining analysis and PCoA. In the SAMOVA analysis (Table S1), *F*_CT_ values increased progressively as *K* was increased in *F. heterostyla*. At *K* = 2, the two identified groups coincided with the two clusters determined by STRUCTURE. The two subclusters within the eastern *F. heterostyla* cluster was also supported by SAMOVA when *K* > 2. For *F. hispida*, the highest *F*_CT_ value was observed at *K* = 2. Under this *K*, populations D-msk and D-cyl were separated from the remaining populations. When *K* > 2, at least one member of the groups contained a single *F. hispida* population, indicating that the group structure was disappearing. The pattern of more poorly resolved genetic relationships and lower spatial genetic structure among *F. hispida* populations than among *F. heterostyla* populations further suggested stronger pollen flow among *F. hispida* populations.

**Fig. 4:**
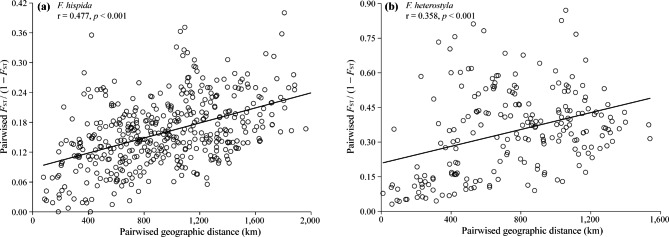
The regression of paired *F*_ST_/(1-*F*_ST_) vs. geographic distance was significant for nSSR data in both *F. hispida* (**a**) and *F. heterostyla* (**b**)

A statistically significant pattern of isolation by distance was observed in both *F. hispida* (r = 0.477, *p* < 0.001, Fig. 4a) and *F. heterostyla* (r = 0.358, *p* < 0.001, Fig. 4b), as well as each intraspecific cluster detected by STRUCTURE (Figs. S5 and S6).

**Fig. 5:**
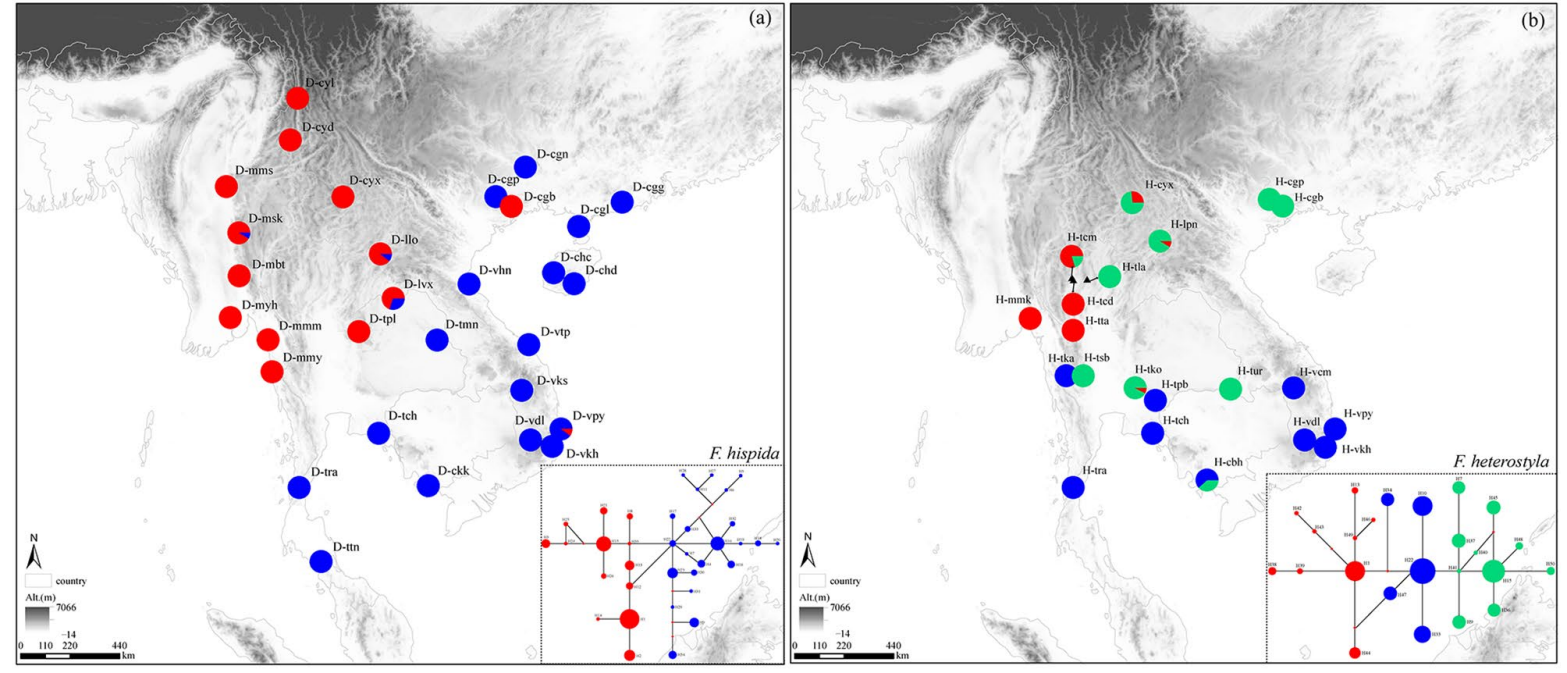
Maps showing the chloroplast DNA haplotype distribution and median-joining network (in the lower right corner) of *F. hispida* (**a**) and *F. heterostyla* (**b**) populations. In the network diagrams, circle size is proportional to the number of individuals with the haplotype, and the nodes with a small red diamond represent intermediate haplotypes

Significant phylogeographic structure signatures (i.e., *N*_ST_ > *G*_ST_) were revealed by cpDNA in both *F. hispida* (*N*_ST_ = 0.798, *G*_ST_ = 0.775, *P* < 0.01) and *F. heterostyla* (*N*_ST_ = 0.826, *G*_ST_ = 0.788, *P* < 0.01). The cpDNA haplotype network demonstrated a decentralized structure in both species. Most haplotypes were localized and differentiated from their connecting haplotypes with only one or two mutation steps. No dominant haplotype was distributed across regions (Figs. S7 and S8). Two distinct haplogroups within *F. hispida* were revealed, roughly in line with the two geographical clusters suggested by STRUCTURE analysis. However, three *F. heterostyla* haplogroups appeared without discernible boundaries between them. The east‒west partition among *F. heterostyla* populations observed when using nSSR was not supported by cpDNA (Fig. 5). Only two haplotypes were shared by the two geographical clusters of *F. hispida* (H4, H15) and *F. heterostyla* (H22, H33). Among the nine interspecifically shared haplotypes, a few, including H1, H10, H15, and H22, occurred in multiple populations and were central nodes of the whole network (H22) or subclades (H1, H10, H15). They were likely derived from the retention of ancestral polymorphisms because of incomplete lineage sorting. Haplotypes, such as H7, H13, H33 and H34, restricted to the geographically adjacent populations of the two figs may result from chloroplast capture, but more evidence is needed.

The cpDNA-based AMOVA tests revealed broader differentiation (*F*_ST_ = 0.821, *p* < 0.001) among *F. heterostyla* populations, with 78.70% of the genetic variation partitioned among populations within clusters, but only 3.67% of the genetic variation was observed between the two geographic clusters. A lower level of differentiation was revealed among *F. hispida* populations (*F*_ST_ = 0.750, *p* < 0.001), with 5.69% of the genetic variation occurring between the two geographic clusters and 69.97% existing among populations within clusters. The nSSR-based AMOVA tests similarly revealed much higher population differentiation in *F. heterostyla* than in *F. hispida*. Significant and broad genetic differentiation (*F*_ST_ = 0.245, *p* < 0.001) was identified among *F. heterostyla* populations, with 14.60% of the genetic variation partitioned between the two geographic clusters and 15.13% observed among populations within clusters. In contrast, much lower differentiation was revealed among *F. hispida* populations (*F*_ST_ = 0.132, *p* < 0.001), with only 3.58% of the genetic variation occurring between the two geographic clusters and 11.12% existing among populations within clusters (Table 2). These results together revealed more strongly limited seed dispersal than pollen dispersal, and higher levels of both seed and pollen flow were observed in *F. hispida* than in *F. heterostyla*. A mysterious exception is that only 3.67% of cpDNA genetic variation occurred between the two *F. heterostyla* geographic clusters, which was lower than that observed in *F. hispida*.

**Table 2:** The analysis of molecular variance (AMOVA) for cpDNA and nSSR data

Data type	Species	Source of variation	Two geographic clusters				Total populations	
			Among clusters	Among populations within clusters	Within populations		Among populations	Within populations
cpDNA	*F. hispida*	df	1	28	288		29	288
		Sum of squares	8.951	104.419	34.649		113.370	34.649
		Variance components	0.02814	0.34588	0.12031		0.36039	0.12031
		Percentage of variation (%)	5.69	69.97	24.34		74.97	25.03
		* F*-statistics	*F*ct = 0.05693	*F*sc = 0.74193	*F*st = 0.75663		*F*st = 0.74972	
	* F. heterostyla*	df	1	19	241		20	241
		Sum of squares	7.576	91.995	20.730		99.572	20.730
		Variance components	0.01793	0.38402	0.08602		0.39345	0.08602
		Percentage of variation (%)	3.67	78.70	17.63		82.06	17.94
		* F*-statistics	*F*ct = 0.03675	*F*sc = 0.81700	*F*st = 0.82373		*F*st = 0.82060	
nSSR	*F. hispida*	df	1	28	600		29	600
		Sum of squares	60.253	358.089	2077.377		418.342	2077.377
		Variance components	0.14519	0.45118	3.46229		0.52624	3.46229
		Percentage of variation (%)	3.58	11.12	85.31		13.19	86.81
		* F*-statistics	*F*ct = 0.03577	*F*sc = 0.11529	*F*st = 0.14694		*F*st = 0.13194	
	* F. heterostyla*	df	1	19	491		20	491
		Sum of squares	274.79	545.369	2268.194		820.16	2268.194
		Variance components	0.95977	0.99484	4.61954		1.49723	4.61954
		Percentage of variation (%)	14.60	15.13	70.27		24.48	75.52
		* F*-statistics	*F*ct = 0.14599	*F*sc = 0.17719	*F*st = 0.29732		*F*st = 0.24477	

Chloroplast DNA sequences identified a higher intercluster gene flow in *F. heterostyla* (*N*m = 7.839) than in *F. hispida* (*N*m = 4.926). While nuclear microsatellites yielded lower intercluster gene flow in *F. heterostyla* (${m_{east \to west}}$: 0.0036; ${m_{west \to east}}$: 0.0028) than in *F. hispida* (${m_{east \to west}}$: 0.0332; ${m_{west \to east}}$: 0.0121).

### Phylogenetic reconstruction and divergence of chloroplast lineages

The results from the BEAST analysis of the psbA-trnH + trnS-trnG dataset indicated that the cpDNA lineages of the two figs began to diversify from the end of the Miocene to the beginning of the Pliocene (*F. hispida*: 5.27 mya, 95% HPD = 2.30–8.42 mya; *F. heterostyla*: 5.65 mya, 95% HPD = 2.03–10.04 mya). Most of the shallow haplotypes of both figs split during the Pleistocene.

**Fig. 6:**
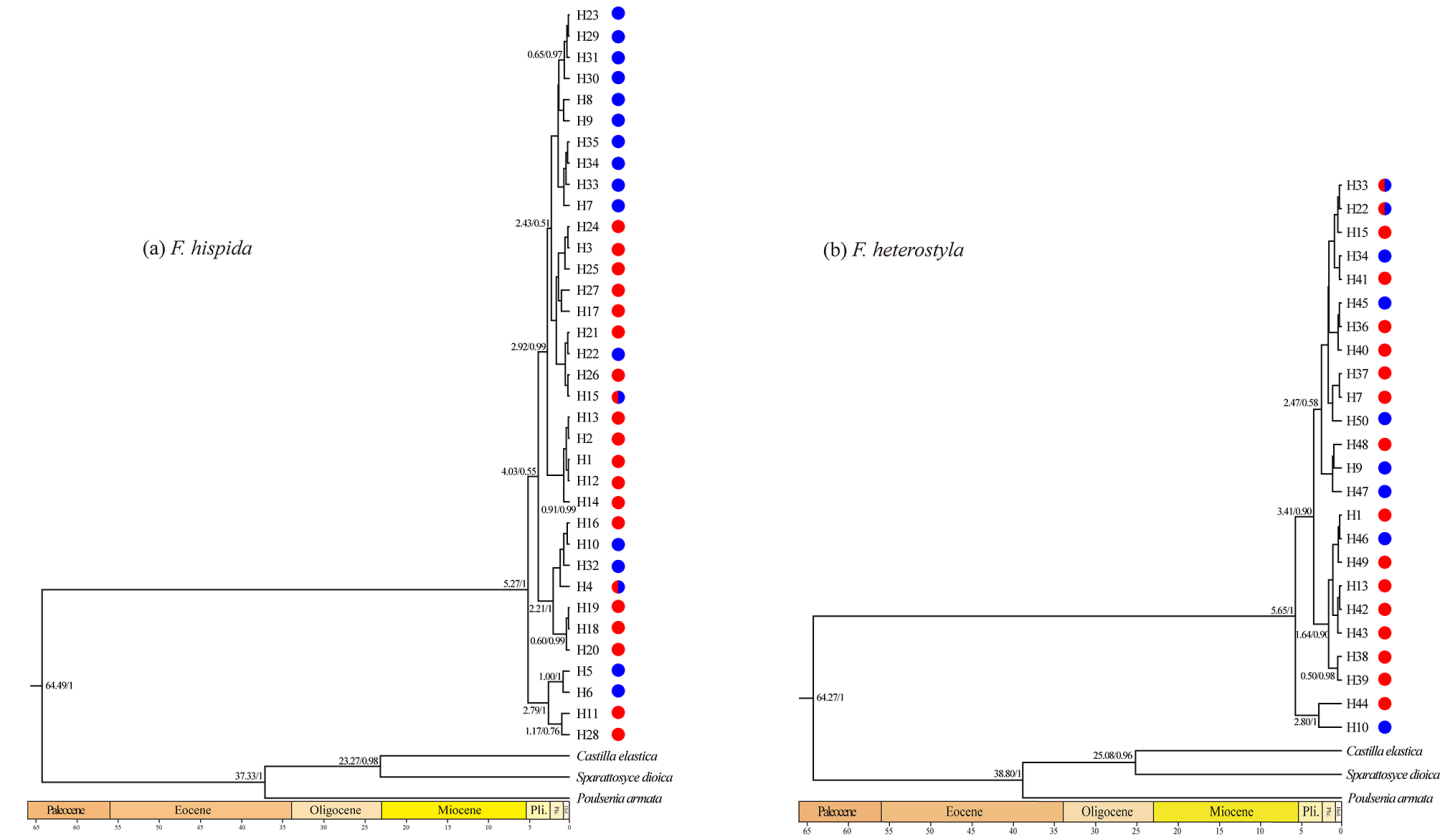
Chronogram of the chloroplast haplotypes of *F. hispida* (**a**) and *F. heterostyla* (**b**) obtained by BEAST analysis of the psbA-trnH + trnS-trnG dataset. The estimated divergence time/Bayesian posterior probabilities (≥ 0.5) are shown beside the nodes. The red- and blue-colored haplotypes indicate that they were located in the western and eastern populations, respectively

Phylogenetic relationships among the shallow haplotypes were not well resolved (posterior probabilities < 0.5) (Fig. 6) because of the recency of divergence. The low level of nucleotide diversity (Table 1) indicated only small nucleotide differences between haplotypes and echoed the lack of phylogenetic resolution. The haplotypes belonging to a geographic cluster revealed by STRUCTURE analysis did not form a monophyletic group, refuting the hypothesis that one divergence event caused the east‒west differentiation pattern derived from nSSR.

### Demographic history

**Fig. 7:**
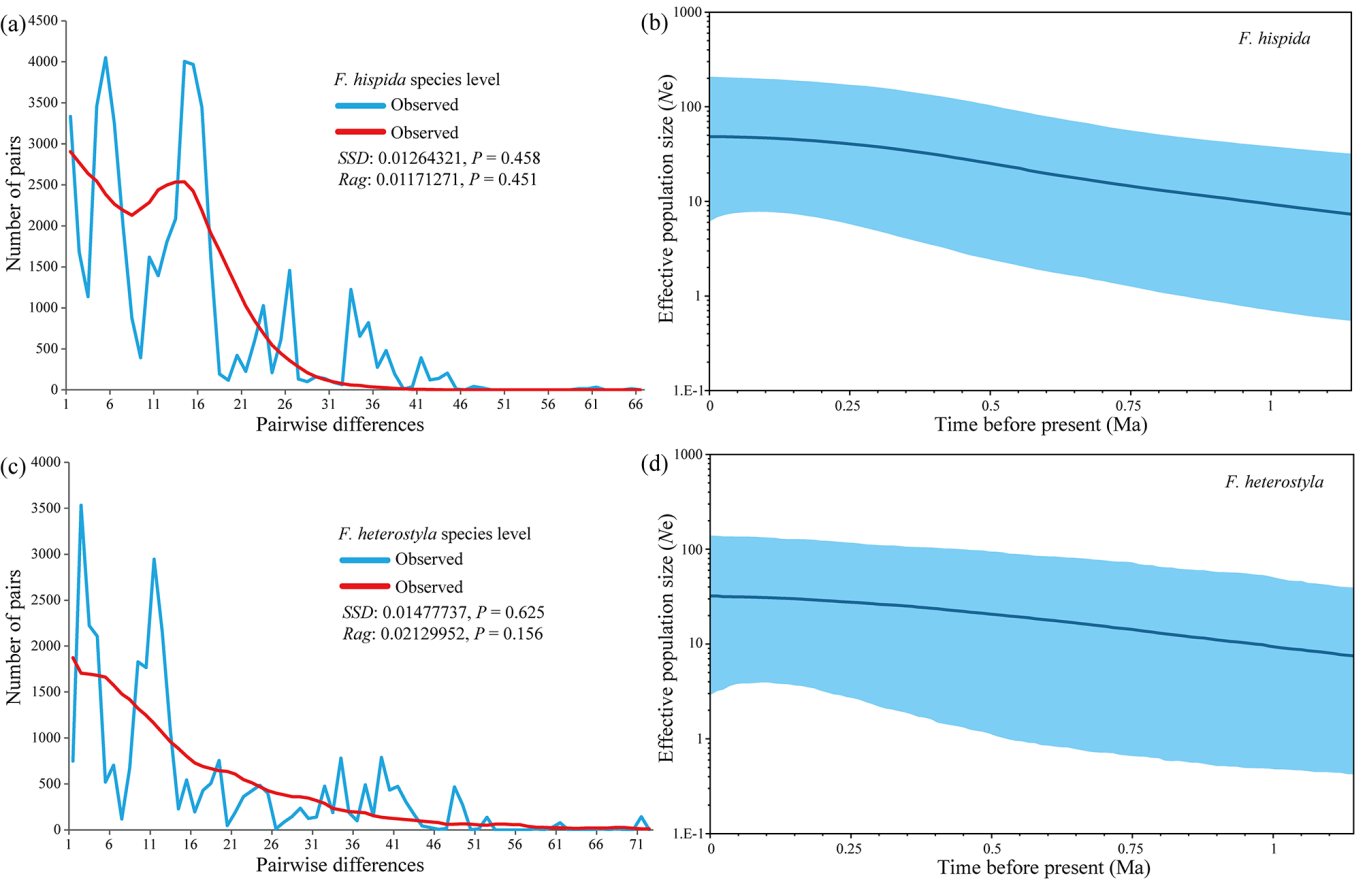
The results of mismatch distribution analysis and Bayesian skyline plots of *F. hispida* (**a**, **b**) and *F. heterostyla* (**c**, **d**) estimated with cpDNA sequences. The thick solid blue line in b and d is the mean estimate, and the area delimited by the light blue broadband represents the highest posterior density 95% confidence intervals for *N*e

**Fig. 8:**
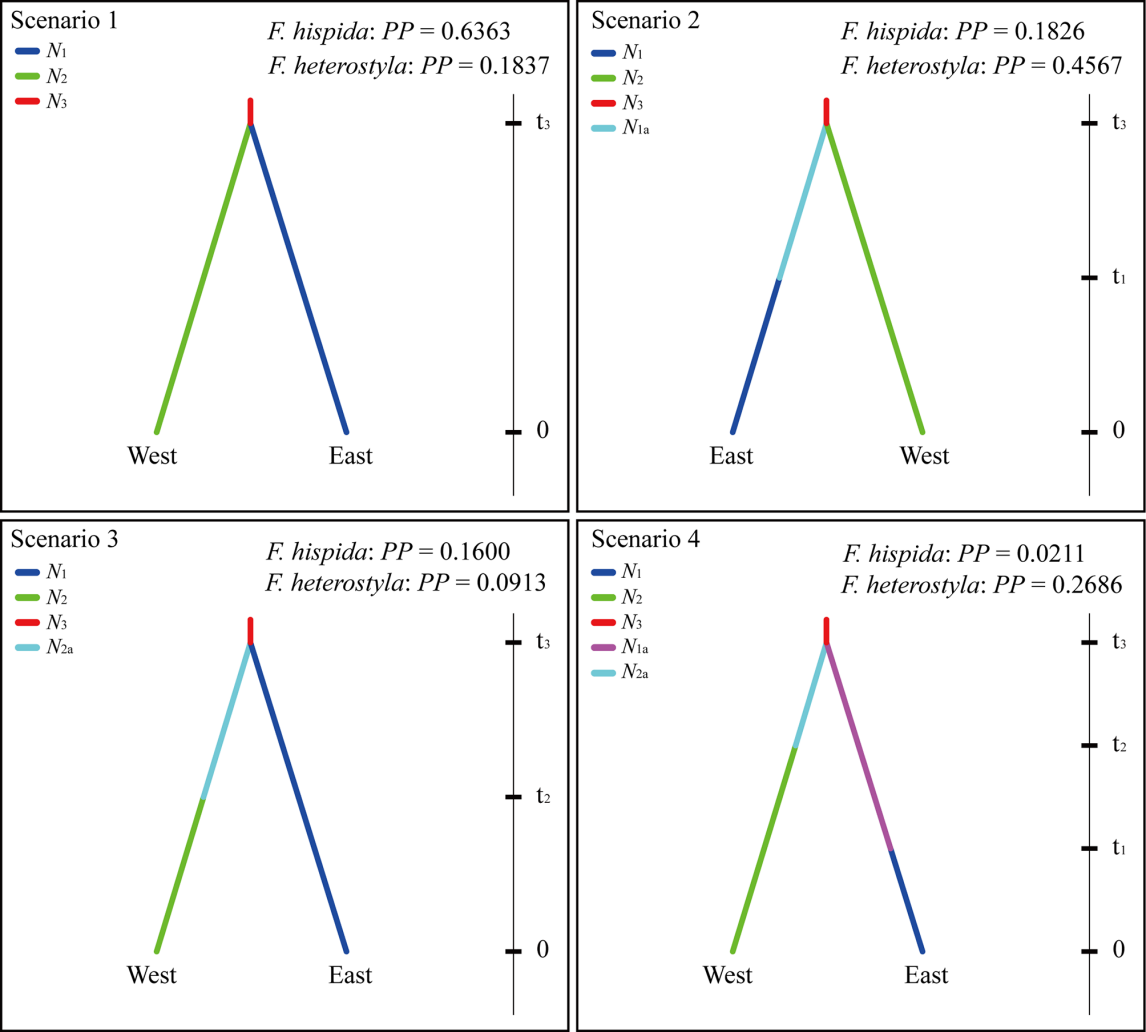
Four scenarios for *F. hispida* and *F. heterostyla* based on Approximate Bayesian Computation. ‘East’ and ‘West’ represent the eastern and western cluster identified by STRUCTURE, respectively. *N*_1_, *N*_1a_: The effective population size of eastern cluster at present and at t_1_, respectively; *N*_2,_*N*_2a_: The effective population size of western cluster at present and at t_2_, respectively; *N*_3_: The effective population size of ancestral populations at t_3_. The time (t_1_, t_2_, t_3_) parameters were estimated in generations; *PP*, posterior probabilities of the scenarios obtained by logistic regression

Neutrality tests with both Tajima’s *D* and Fu’s *F*_S_ statistics yielded nonsignificant negative values for *F. hispida* (Tajima’s *D* = *-*0.843, *P* = 0.183; Fu’s *F*_S_ = -4.525, *P* = 0.089) and *F. heterostyla* (Tajima’s *D* = *-*0.181, *P* = 0.426, Fu’s *F*_S_ = -0.444, *P* = 0.492), suggesting that no significant population expansion has occurred in the recent past. Likewise, no sudden population expansion was obviously supported by multimodal patterns of mismatch distributions, with nonsignificant *SSD* and *Rag* values in both species (Fig. 7a, c). Nevertheless, the BSP results showed that both figs experienced weak population expansion for a long time (Fig. 7b, d). Analysis with DIYABC identified Scenarios 1 and 2 as most highly supported for *F. hispida* (*PP* = 0.6363) and *F. heterostyla* (*PP* = 0.4567), repectively. The population divergence times (t_3_) between western and eastern clusters were estimated as 1250 (95%HPD: 332–2980) and 4910 (95%HPD: 1300–9620) generations for *F. hispida* and *F. heterostyla*, respectively. If assuming a generation time of ~ 5 years for these two small trees, the estimated t_3_ was 7.25 kya (95%HPD: 1.66–14.90 kya) and 24.55 kya (95%HPD: 6.50–48.10 kya) for *F. hispida* and *F. heterostyla*. After divergence, Scenario 1 suggested no significant population expansion for both eastern and western *F. hispida* clusters. While, Scenarios 2 supported the expansion of eastern *F. heterostyla* cluster (*N*_1_ = 5250, *N*_1a_ = 1350) after approximately 1380 (t_1_) generations. The ancestral populations of both *F. hispida* (*N*_3_ = 1050) and *F. heterostyla (N*_3_ = 1430) were estimated to be small than present (Fig. 8; Table S2), suggesting the population expansion at species level.

### Ecological niche modeling and recent expansions

Nine bioclimatic variables were selected to model the ecological niche of *F. hispida* (Table S3, Fig. S9). According to the results from both analyses of variable contributions and the jackknife test (Fig. S10), minimum temperature of the coldest month (Bio6), precipitation of the driest month (Bio14) and isothermality (Bio3) were the three most important variables and contributed the most to the prediction of suitable habitats of *F. hispida.* The models were validated using AUC values, with all models showing an AUC > 0.80 (LIG: 0.845; LGM, 0.835; MIH, 0.844; present 0.851; future 2070, 0.849) and indicating the high accuracy of the model.

**Fig. 9:**
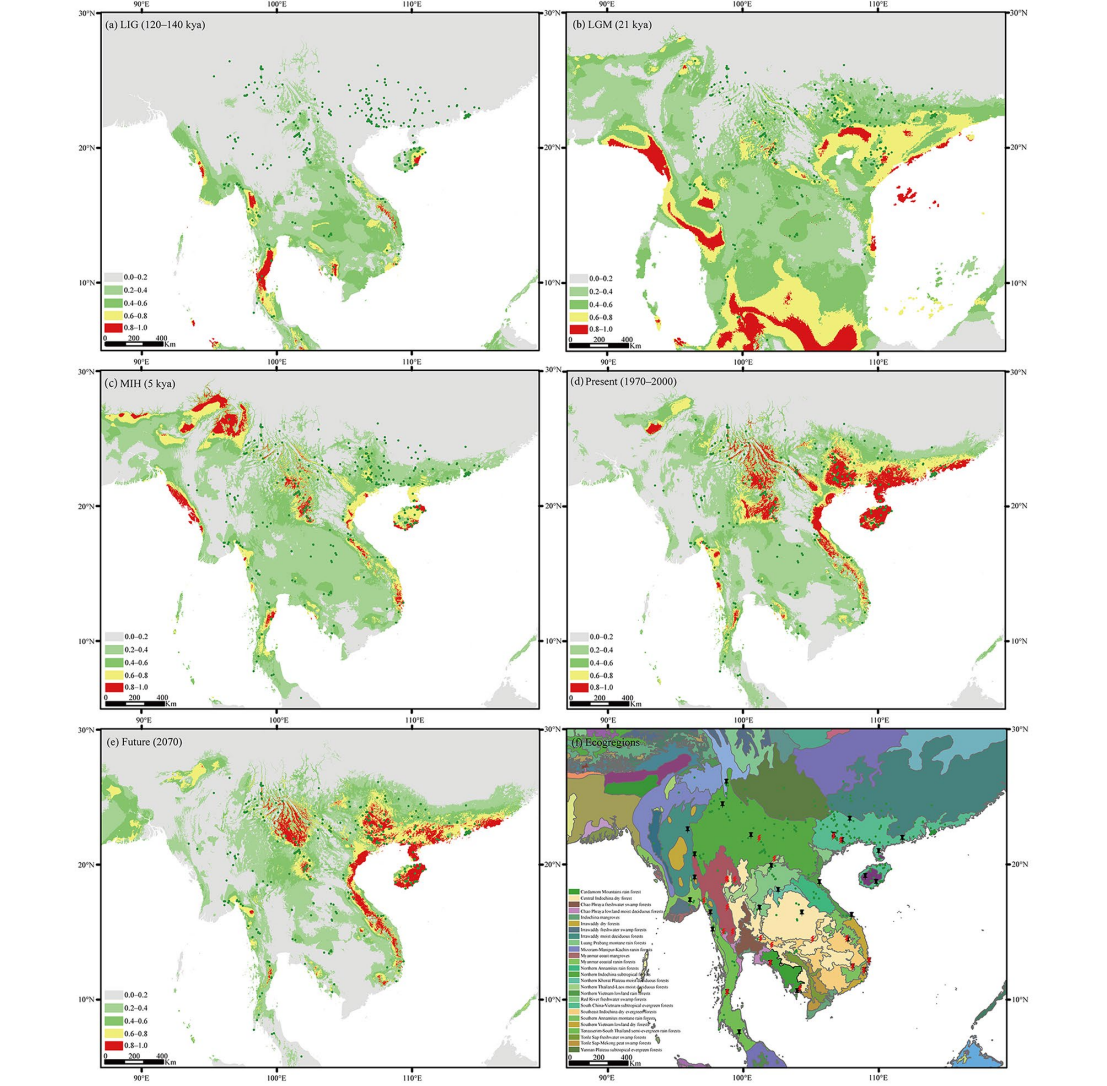
Potential distributions of *F. hispida* predicted using MaxEnt based on nine bioclimatic variables representing the LIG (**a**), LGM (**b**), MIH (**c**), present (**d**) and future (**e**) climatic conditions. Warmer colors denote areas with a higher probability of presence. Green dots show the extant occurrence record points of *F. hispida*. Terrestrial ecoregions of the Indo-Burma region are colored in (**f**). The black needles and red strikes indicate the population samples of *F. hispida* and *F. heterostyla*, respectively

Past, present, and future predictions of suitable habitat for *F. hispida* are shown in Fig. 9. The predicted distribution in the present was largely consistent with known occurrences. Two centers with high habitat suitability appeared in northern Indo-Burma: one ranged from central Vietnam to southern China, and the other ranged from northern Thailand to southwest Yunnan of China. Compared with the present, the predicted species distribution shrinked severely during the LIG and expanded substantially during the LGM. High LIG and LGM habitat suitability mainly occurred in the coastal areas of Indo-Burma; nevertheless, the high LGM habitat suitability are currently mostly submerged. During the MIH, high habitat suitability substantially decreased compared with that in the present and LGM periods, but it increased compared with the LIG period. Future projections for 2070 suggested slight contraction of highly suitable and subsuitable habitats (colored light green in Fig. 9a-e).

Vast areas of Cambodia, central and southeast Thailand, and neighboring Laos that covered with dry forests (Fig. 9f) and high-altitude mountain regions of northern Laos and Vietnam were not suitable for *F. hispida* during all the examined periods. The subtropical evergreen forests, montane forests, and coastal and montane rainforests were predicted to have high habitat suitability. High-altitude mountains in northern Laos largely separated the two high-habitat suitability centers. All 396 present-day occurrences were recorded at altitudes below 1,200 m, suggesting that altitude is an important limiting factor for the survival of *F. hispida*.

## Discussion

We conducted a comparative phylogeographic analysis of dioecious *F. hispida* and *F. heterostyla* across the Indo-Burma hotspot. Both species were revealed to have strong phylogeographic structure and similar spatial distributions of genetic diversity. In particular, a conspicuous east‒west differentiation pattern was disclosed firstly for the Indo-Burmese plants. Whereas, interspecific dissimilarities at fine-scale genetic structure and asynchronized historical dynamics of east-west differentiation were observed, which can be attributed to the differences in pollen and seed dispersal syndromes.

### Spatial distribution of genetic diversity and conservation implications

Both species showed similar spatial distributions of genetic diversity and displayed high levels of population-specific cpDNA haplotypes and nSSR alleles. The northern Indo-Burmese populations, especially those distributed in humid subtropical evergreen forests in low-altitude mountain areas, were revealed with high genetic diversity and frequencies of private alleles (Table 1, Fig. S2). These areas were identified as long-term climatically stable refugia for East Asian relict plants [55] and also showed high habitat suitability from LGM to future decades for the two focal *Ficus* plants (Fig. 9); thus have and will continue to serve as potential climate refugia contributing to the conservation of biodiversity and should be considered conservation priority areas. The complex topography, geomorphology, and climatic history and resulting extremely diverse landscapes, climatic regimes and isolated habitats drive the endemism of Indo-Burmese flora and fauna [23, 56–58] and may have contributed to the high numbers of population-specific cpDNA haplotypes and nSSR alleles observed in *F*. *hispida* and *F*. *heterostyla*.

### The East‒West differentiation pattern

A conspicuous east‒west differentiation pattern was revealed in both *F. hispida* and *F. heterostyla*. The lineage diversification and divergence of two figs were estimated to occur mainly from the Pliocene to Pleistocene (ca. 5.3 mya–12 kya), when the geographic features, continental outline and mountains were in place and relatively stable [59, 60]. Thus, geological processes (e.g., tectonic movement, orogenesis) are unlikely to have greatly affected the present phylogeographic structure. However, the present-day characteristic topography, geomorphology and monsoon climate of Indo-Burma created by the complex geological and climatic history, together with biotic factors, jointly shaped the phylogeographic structure of the two figs.

Indo-Burma is characterized by many meridionally oriented mesoscale mountain ranges, which are expected to impede gene exchange between populations on either side of the mountains. The two studied species were recorded to grow at low altitudes [39, 61]. All 396 *F. hispida* occurrences and 199 *F. heterostyla* occurrences we collected [62] were recorded at altitudes below 1,200 m. Moreover, the tiny, short-lived pollinating wasps are sensitive to temperature and humidity [63–65]. Increasing altitude with decreasing temperature and humidity will reduce the survivability of wasps and create barriers for trans-alpine pollen dispersal. For example, the north‒south-running mountain ranges stretching from Yunnan of China to southern Burma likely hindered gene exchange and separated the six western populations (D-mms, D-myh, D-msk, D-mbt, D-cyd, D-cyl) from the eastern *F. hispida* populations. While, the Three Pagodas Pass could have served as a corridor of gene exchange between populations on both sides of the Tenasserim Hills formed by a series of north–south-trending mountains. This hypothesis is supported by the genetic affinities between *F. heterostyla* populations H-mmk and H-tka.

Indo-Burma is dominated by a tropical monsoonal climate with apparent intraseasonal variability. Summer southwestern and winter northeastern monsoons prevail alternately across this region [66, 67]. Winds greatly encourage the long-distance dispersal of pollinating wasps, as suggested by both observational [47, 68–72] and genetic [11, 48, 49, 53, 73] studies. For example, southwesterly monsoon winds could have increased the rate of wasp-mediated pollen flow in a northeasterly direction across the eastern and southeastern Asian ranges of *F. hirta* [53]. Both *F. hispida* and *F. heterostyla* bear figs year-round [74–76], and their pollinators experience southwestern winds in summer, shifting to northeastern winds in winter. A largely increased or decreased genetic proportion of one geographical cluster mixed into another cluster in the southwesterly or northeasterly direction observed in *F. hispida* coincided with the monsoonal directions. In addition, the genetic affinities of populations isolated by oceans in the southwesterly direction, including Vietnamese populations D-vtp and D-vhn, Hainan Island populations D-chc and D-chd, and Guangdong populations D-cgl and D-cgg (Fig. 1), suggested the wind-assisted transoceanic dispersal of pollinating wasps of *F. hispida*. Nonetheless, wasps of *F. heterostyla* are unlikely to travel via winds due to the geocarpic figs.

The tropical monsoonal climate determines the seasonal variation in rainfall and temperature in most Indo-Burma regions [60, 77, 78]. Nevertheless, rainfall and its seasonality are more significant in determining the Indo-Burma vegetation than temperature variation [58, 60]. Due to the meridionally oriented Arakan Range in Myanmar and the Annamite Range in Vietnam serving as natural barriers in the western and eastern coastal regions, respectively, precipitation is mainly concentrated along the western and eastern coasts and generally decreases inland (such as on the Thai-Lao Dry Plateau) [60, 77]. In the context of dynamic monsoon circulation patterns, the complex topography and geomorphology, landscape and elevation changes have further increased the rainfall variation at regional and local levels [57, 79]. The relatively dry regions that received lower precipitation (< 2000 mm) and extended dry seasons (5 to 7 months) were dominated by savannah vegetation and occurred mosaically across Indo-Burma [80, 81]. Such vegetation likely experienced multiple extended periods during the Pliocene and Pleistocene with global intensified cooling and aridification and then reached a climax at the LGM [81–83]. Dry savannahs, perhaps even forming a continuous north‒south belt, dominated northern, central and eastern Thailand during glacial periods [82, 84, 85]. The ENM analysis also showed that vast areas of central Indo-Burma are not suitable for *F. hispida* (Fig. 9) and *F. heterostyla* [62], where are currently dominated by a tropical savanna climate [86]. This continuous or disconnected dry belt would have operated as a barrier to east‒west gene exchange of rainforest species [87], as well as the two studied figs preferring moist habitats.

*F*. *hispida* is mainly found along rivers or in swamp edges. The figs of *F. heterostyla* are located on rooting stolons near or under the soil. Adventitious roots will often grow from the leafless rooting stolons (Fig. S1) and may facilitate water absorption. During our field observation and manipulative experiments, we found that figs of *F. heterostyla* show a high level of abortion before maturity during the dry season (our unpublished data), suggesting that soil humidity profoundly influences the development of fig fruits. Thus, precipitation could be one of the most important factors affecting the distribution and genetic structures of the two studied figs, especially for *F. heterostyla*. The differentiation of the two emerging subclusters within the eastern cluster of *F. heterostyla* is likely attributable to the unsuitable arid zone between the two subclusters (Figs. S5 and 8). However, this pattern was not observed in *F. hispida* and suggested that droughts constrained *F. heterostyla* even more. In addition, droughts greatly reduce the survivability of pollinating wasps [64, 88–91]. Thus, with the additive damage of cooling, it is unlikely for the short-lived, tiny wasps to cross the dry belt.

### Contrasting phylogeographic structure between species

Interspecific dissimilarities at fine-scale genetic structure were discovered and mainly ascribed to the interspecific differences in seed and pollen dispersal syndromes. Lower level of population differentiation and higher level of intercluster differentiation in *F. hispida* than in *F. heterostyla* were revealed by chloroplast data. Fig fruits are consumed by highly diverse frugivore assemblages, which in turn serve as seed dispersal agents [92]. Frugivorous bats are the main fruit consumers of *F. hispida*, including the lesser short-nosed fruit bat (*Cynopterus brachyotis*), the greater short-nosed fruit bat (*Cynopterus sphinx*), and Leschenault’s rousette (*Rousettus leschenaultii*) [93–95]. In contrast, ground-foraging animals, such as rodents and deer [96], are the potential consumers of geocarpic *F. heterostyla* figs. Frugivorous bats are mobile seed dispersers, as shown by the lower chloroplast differentiation among *F. hispida* populations than among *F. heterostyla* populations. However, frugivorous bat movement is largely determined by fruit resources [97–99]. The savannah belt or a band of open vegetation in Indo-Burma is supposed to be inhospitable for frugivorous bats due to the scarcity of food and roosts, as well as increasing exposure to predators (e.g., owls, hawks) [100–102], retarding the east‒west seed dispersal of *F. hispida*. Nevertheless, this dry savannah vegetation was not as inhospitable to ground-dwelling animals and may have even served as a north‒south migration corridor (connecting Indo-Burma to Java) for open vegetation-adapted species during Pliocene-Pleistocene glacial periods, such as *Macrotermes* [103], mammals [104], and early humans [105, 106]. Thus, the seed dispersal between eastern and western *F. heterostyla* clusters mediated by ground-dwelling animals was revealed to be much stronger than that of *F. hispida*.

Much lower levels of population and intercluster differentiation in *F. hispida* than in *F. heterostyla* was revealed by nSSR data, suggesting stronger pollen flow in *F. hispida*. Similar to the prostrate shrub *F. tikoua* [51], figs of *F. heterostyla* lie close to the ground or are even partially buried by soil. This geocarpic pattern means that floral volatile attractants for pollinators released by receptive figs of their host *Ficus* are likely to be highly localized and close to the ground; thus, long-distance dispersal of its pollinators is poorly suited to finding the figs of these species [47, 51]. Instead, the pollinators of *F. heterostyla* may stay close to the ground where the potential receptive figs are to be found. The observed genetic homogeneity in the populations, subclusters or clusters of *F. heterostyla* may be partly explained by the prevention of local divergence due to limited pollen flow. *F. hispida* produces larger crops and bears figs positioned on branchlets arising from main branches or trunk above the ground. A larger crop will produce a larger volatile plume and should be more easily detected and responded to by distant pollinators [47]. In addition, flight of pollinators of *F. hispida* above the ground may be aided by airflow and will extend the pollinating distance.

### Demographic dynamics

Combining multiple methods and both cpDNA and nSSR data, population expansion was believed to have occurred in both species, although the signal of rapid expansion was not detected. Range expansion in obligate mutualisms involving free-living organisms requires the successful range extension of independently dispersing partners [53, 107]. The successful colonization and reproduction of host figs in a new location also depends on the successful population establishment of associated pollinating wasps, while pollinator absence often appears to limit the range expansion of host Figs.  [38, 53, 108]. Pollinating wasps associated with dioecious figs are often short in dispersal distance, especially for geocarpic species such as *F. tikoua* [51] and *F. heterostyla.* The highly restricted pollen and seed dispersal likely limited the expansion of *F. heterostyla*, supporting that ‘species-specific pollination appears to be more a limitation than a help for range expansion’, as observed in *F. carica* [108]. *F. hispida* showed slightly higher seed flow and much higher pollen flow than *F. heterostyla*; thus, the expansion of *F. hispida* likely slowed more strongly by limited seed dispersal than pollinator dispersal. From the perspective of pollen and seed dispersal, the expansion of *F. heterostyla* was expected to be more strongly limited than that of *F. hispida*, which was supported by the neutrality test. Although the Tajima’s *D* and Fu’s *Fs* values were nonsignificantly negative, these two values for *F. heterostyla* were greater than those for *F. hispida*, hinting at a greater excess of rare alleles in *F. hispida* that resulted from population expansion. Furthermore, the preferences and demands for moist habitats may further limit expansion, especially for *F. heterostyla*. Due to the increasing human-caused loss of vertebrates, the spread of fig fruits via vertebrates and the expansion of *Ficus* species will face increasing threats. Future projection for 2070 by ENM analysis also suggested reduced habitat suitability across Indo-Burma for *F. hispida* (Fig. 9) and *F. heterostyla* (see Fig. 3 in [62]).

The DIYABC analysis based on nSSR revealed that eastern and western *F. heterostyla* clusters splitted before the LGM, while the east-west differentiation pattern of *F. hispida* shaped after the LGM. It echoed that *F. heterostyla* and its pollinating wasps are more sensitive to climate changes, and thus the intercluster pollen flow is more susceptibles to climatic disturbance. However, even in the LGM, there may be considerable gene flow between eastern and western *F. hispida* clusters. The ENM analysis suggested gradually reduced habitat suitability for *F. hispida* from central Thailand to Cambodia after the LGM, supporting the increasing differentiation between eastern and western *F. hispida* clusters aftern the LGM.

## Conclusions

We confirm hypothesized predictions that interactions between biotic and abiotic factors largely determine the patterns of genetic diversity and phylogeographic structure of Indo-Burmese plants. The characteristic south–north-oriented high mountains and monsoon climate, especially the variation in precipitation caused by monsoon climate, as well as the preferences and demands for moist habitats of the plants, most likely resulted in the east‒west differentiation pattern shown by *F. hispida* and *F. heterostyla*, which is potentially generalizable to some other Indo-Burmese plants. Species-specific features, especially those involving pollen and seed dispersal, are responsible for the idiosyncratic patterns among codistributed organisms, as we observed in the two studied *Ficus* species. The low-altitude mountain areas in northern Indo-Burma with high genetic diversity and high habitat suitability may have and continue to serve as potential climate refugia. These results provide insights into the conservation of Indo-Burmese biodiversity and will facilitate targeted conservation efforts.

## Materials and methods

### Study species and sampling

*Ficus hispida* is a shrub or tree up to 15 m and is widely distributed over tropical Asia and Australasia. It is mainly found along rivers or in swamp edges and is common in secondary growth as a pioneer species. It is predominantly cauliflorous or sometimes produces syconia on short fig-bearing branchlets arising from main branches or trunk [39, 61] (Fig. S1a–e). The fig is pollinated by the agaonid wasp *Ceratosolen marchali-solmsi* and will turn pale yellow at maturity, which is mainly consumed by bats [104, 105] to disperse the seeds. *F. heterostyla* is a shrub or small tree that grows up to 5(–8) m tall and was proposed as distinct from *F. hispida* by Berg and Chantarasuwan [61] mainly because of differences in fruiting position and hair color on leaves and stems. It grows under the forest canopy and often in secondary growth at low altitudes, ranging from Xishuangbanna of China to Vietnam. Figs are located in rooting stolons near or under the soil and will become orange red to brownish at maturity [61, 76] (Fig. S1f–j). It is pollinated by an undescribed *Ceratosolen* wasp, and the geocarpic figs could limit wasp dispersal [109]. Although there are no detailed records, ground-foraging animals are probably the main consumers according to fruit positions and traits.

These two dioecious figs are phylogenetically closely related [44] and co-occur in Indo-Burma. Leaf samples were collected from 326 individuals of 30 *F. hispida* populations and 276 individuals of 21 *F. heterostyla* populations (Table 1; Fig. 1) for DNA extraction, covering southern China, Myanmar, Thailand, Laos, Vietnam and Cambodia (i.e., Indo-Burma).

### DNA extraction, cpDNA amplification and sequencing

Genomic DNA of individual samples was extracted using the Tiangen Plant Genomic DNA Kit (Tiangen Biotech, Beijing, China). Two cpDNA intergenic regions, psbA-trnH and trnS-trnG [110], were chosen for amplification. The amplified products were bidirectionally sequenced by the Beijing Genomics Institute (Shenzhen, China). All forward and reverse strands were edited and assembled using Sequencher 4.5 (GeneCodes, Ann Arbor, Michigan, USA). The sequences were aligned and then adjusted manually using BioEdit 7.0.9.0 [111]. A matrix of combined sequences for psbA-trnH and trnS-trnG was constructed.

### Nuclear microsatellite amplification and genotyping

We initially screened a set of 19 loci for the two focal figs, and fourteen of them (3-N173, 4-101, 5-N108, 6-N104, 7-N245, 19-N530, 20-N291, 21-N197, 23-N724, 26-N180, 28-N247, 29-N105, 30-N457 and 32-N125) were selected for amplification and genotyping according to the amplification protocols described in Li et al. [109]. Post-PCR products were analyzed by capillary electrophoresis on an ABI 3730XL DNA analyzer (Applied Biosystems, Foster City, California, USA) with the GeneScan 500 ROX Size Standard. Microsatellite fragment sizes were determined using GeneMapper version 3.2.

### Genetic diversity

CpDNA haplotypes were distinguished using DnaSP v5 [112] on the basis of nucleotide and indel differences. The number of haplotypes (*H*), nucleotide (*π*) and haplotype (*H*_d_) diversity, were calculated using the same program. For the nSSR data, classical indices of genetic diversity, including mean number of alleles (*N*_a_) and private alleles (*PA*) per locus, observed (*H*_O_) and expected heterozygosity (*H*_E_), were calculated using GenAIEx v6.5 [113]. Subsequently, these molecular diversity indices were used to show the geographic pattern by using the inverse distance weighted (IDW) interpolation function implemented in ArcGIS v10.3. IDW assumes that points close to each other are more relevant than those that are more distant and are weighted more closely to the predicted position than those with farther distances.

### Phylogeographic structure

For the cpDNA dataset, we used PERMUT 2.0 [114] to test the occurrence of phylogeographic structure signatures by comparing two measures of population differentiation, *N*_ST_ and *G*_ST_, based on 1,000 random permutations. The genealogical relationships among cpDNA haplotypes were estimated by the median-joining method implemented in NETWORK 10.2.0.0 [115]. Individual indels were treated as single mutation events.

For the nSSR dataset, a pattern of isolation by distance was assessed using the Mantel test in GenAlEx 6.5 with 9,999 permutations to evaluate the correlations between pairwise genetic (*F*_ST_/(1–*F*_ST_)) and geographic distances. To estimate the genetic affinity of the studied populations, principal coordinate analysis (PCoA) was conducted with GeneAIEx 6.5 based on Euclidean distance. An unrooted neighbor-joining tree was constructed using POPULATIONS v1.2.31 [116] based on the chord distance (*D*_*C*_) of Cavalli-Sforza & Edwards [117], which is preferred for microsatellite data [118]. Bootstrap analysis was performed with 1000 replications, and the tree was visualized by Figtree v1.4.4. Bayesian genetic clustering of individual genotypes was implemented in STRUCTURE v2.3.4 [119]. We employed a model with admixture, with a burn-in period of 100,000 and a run length of 1,000,000 iterations, varying *K* from *K* = 1 to *K* = 10. For each value of *K*, ten runs were performed. The STRUCTURE HARVESTER online program [120] was used to detect the optimal *K* value using the Evanno method [121]. CLUMPP 1.1.2 [122] was used to summarize the membership coefficients into clusters, and the CLUMPP outputs were visualized in DISTRUCT 1.1 [123]. In addition, a spatial analysis of molecular variance (SAMOVA) was performed with SAMOVA 2.0 [124] to identify groups of populations presenting spatial genetic homogeneity. This program finds the best number of geographic groups (*K* value) by maximizing *F*_CT_ value between *K* groups of geographically adjacent populations. The *K* was set from 2 to 10 and 1000 annealing simulations were performed for each *K*.

According to the results of clusters from STRUCTURE, a hierarchical analysis of molecular variance (AMOVA) was performed in Arlequin 3.5 [125] to quantify the differentiation among clusters, among populations within clusters, and within populations based on both cpDNA and nSSR datasets. The significance of statistical indices was tested with 10,000 permutations. Furthermore, genetic differentiation coefficient (*GammaSt*) among clusters was calculated using DnaSP v5 and gene flow was calculated according to the equation *N*m = (1/*GammaSt* − 1)/2 [126] for cpDNA data. Contemporary migration among clusters was estimated following a Bayesian approach implemented in BayesAss v3.0.4 [127] for microsatellites. We ran the program with 10 million iterations, a burn-in run of 1 million and interval sampling of 100. Ten runs with different initial seeds were performed to check for consistency of results and trace plots were examined using Tracer v1.7.1 [128].

### Phylogenetic reconstruction and divergence of chloroplast lineages

Phylogenetic relationships of cpDNA haplotypes of the two fig species were reconstructed with the program BEAST v1.8.1 [129]. The best-fit evolutionary model and gamma rate heterogeneity determined using the Akaike Information Criterion for each chloroplast fragment were selected using PAUP* v4.0b10 [130] and Modeltest 3.7 [131, 132]. Three taxa (*Castilla elastica*, *Poulsenia armata*, *Sparattosyce dioica*) of tribe Castilleae (sister to tribe Ficeae comprising only the genus *Ficus*) were chosen as outgroups (Table S4).

Input files were created using the program BEAUti v1.8.1. The combined dataset was partitioned by locus, and model parameters were unlinked across partitions. The HKY + I model for psbA-trnH and trnS-trnG of *F. hispida* and psbA-trnH of *F. heterostyla* and HKY + G model for trnS-trnG of *F. heterostyla* were suggested by Modeltest. The tree prior model was set using a coalescent approach assuming a constant population size. Based on age constraints inferred by multiple fossil records across family Moraceae and the combined dataset of chloroplast and nuclear sequence fragments, the mean divergence time between tribes Ficeae and Castilleae ranged from 57.8 [50.1–65.8] mya [133] to 72.0 [59.6–88.2] mya [134]. Here, 57.8 and 72.0 mya were set as the lower and upper divergence times between *Ficus* and its sister tribe Castilleae. A normal distribution was specified for the prior tree root age with a mean value of 64.9 mya and a standard deviation including the age estimates for the divergence between *Ficus* and tribe Castilleae. A lognormal relaxed clock model of rate change was applied. We ran MCMC simulations for 2.0 × 10^8^ generations, sampling every 10,000 generations. Convergences were checked using Tracer v1.7.1 to ensure that the value of the effective sample size (ESS) for each statistic was above 200. The condensed tree was drawn using TreeAnnotator v1.8.1 with a 10% burn-in and visualized using Figtree v1.4.4.

### Demographic history

Inference of historical processes was performed using neutrality and mismatch distribution tests. For the neutrality test, Tajima’s *D* considering the frequency of mutations [135] and Fu’s *F*_S_ [136] based on the cpDNA haplotype distribution were calculated. The mismatch distribution test was used to assess whether the observed distribution of pairwise differences matched expectations under the sudden demographic expansion and spatial-demographic expansion models. A smooth unimodal distribution of the observed differences is taken as evidence of a recent population expansion, whereas a “ragged” multimodal distribution is expected under demographic equilibrium or genetic subdivision. The sum of squared differences (*SSD*) and Harpending’s raggedness index (*Rag* [137],) were employed to assess whether the model worked well for the observed and expected mismatch distributions, using 1000 bootstrap replicates [138]. These analyses were all performed in Arlequin v3.5. We also retraced the demographic history of focal figs through a Bayesian skyline plot (BSP) coalescent model to further infer the changes in effective population size over time in BEAST v1.8.1. The BSP was constructed with the same settings as in the previous BEAST analysis except that the prior setting was changed to Coalescent Bayesian Skyline.

We further used ABC simulations in DIYABC v2.0 [139] to determine the historic process involved in the settlement of clusters identifed by STRUCTURE based on the 14 neutral SSR loci. Four possible demographic scenarios were compared between the eastern and western clusters (see section “Results”) of *F. hispida* and *F. heterostyla* (Fig. 8), respectively. For the historical models, priors were set by default. We ran one million simulations for each scenario and compared them by estimating posterior probabilities using logistic regression.

### Ecological niche modeling (ENM)

ENM was carried out in MaxEnt 3.4.1 [140, 141] to predict the potential distribution range of *F. hispida* in the Last Interglacial (LIG: ca.120–140 kya before present), Last Glacial Maximum (LGM: ca. 21 kya before present), Mid-Holocene (MIH: ca. 5 kya before present), present (1970–2000) and future (2070). We recently performed ENM analysis of *F. heterostyla* [62]. In total, 396 occurrence records of *F. hispida* across Indo-Burma were obtained from the GBIF (Global Biodiversity Information Facility, https://www.gbif.org), the CVH (Chinese Virtual Herbarium, https://www.cvh.ac.cn) and our field expeditions. The 19 bioclimatic variables corresponding to the five focal periods were downloaded from the WorldClim database (http://www.worldclim.org). Pairwise correlations of the 19 variables were tested to avoid variable multicollinearity and model overfitting. Variables with Pearson correlation coefficients of |r| ≤ 0.8 were used for subsequent analyses. 75% of the occurrence records were used as training data, and 25% were used as test data in 10 replications. MaxEnt outputs represent the habitat suitability, ranging from 0.0 to 1.0, in Cloglog format. The accuracy of each model prediction was evaluated statistically using the area under the receiver operating characteristic (ROC) curve [142]. The AUC ranges from 0 to 1, where a score above 0.7 is considered an indicator of good model performance [143]. The importance of each climatic variable for explaining the potential distribution was determined by jackknife resampling of the training and test gains. In addition, we matched the potential distribution to the ecoregions to determine which ecoregions were suitable for *F. hispida.* The map of ecoregions was derived principally from patterns of rainfall, temperature, geological history, broad vegetative patterns and expert opinion on community distributions [144, 145].

## Electronic supplementary material

Below is the link to the electronic supplementary material.

Supplementary Material 1

Supplementary Material 2

## Data Availability

Chloroplast sequence of individual haplotype are deposited in NCBI (accession numbers, psbA-trnH: OQ296142–OQ296191; trnS-trnG: OQ296192–OQ296241). Nuclear microsatellites genotyping data needed to replicate this study are provided as supplementary information files.
