# Supplementary material for: East‒West genetic differentiation across the Indo-Burma hotspot: evidence from two closely related dioecious figs
Source: BMC Plant Biol. 2023 Jun 16;23:321. doi: 10.1186/s12870-023-04324-6 (PMC10273766; doi:10.1186/s12870-023-04324-6)
Supplement: Supplementary file 1 — Supplementary Material 1 [file 12870_2023_4324_MOESM1_ESM.docx]

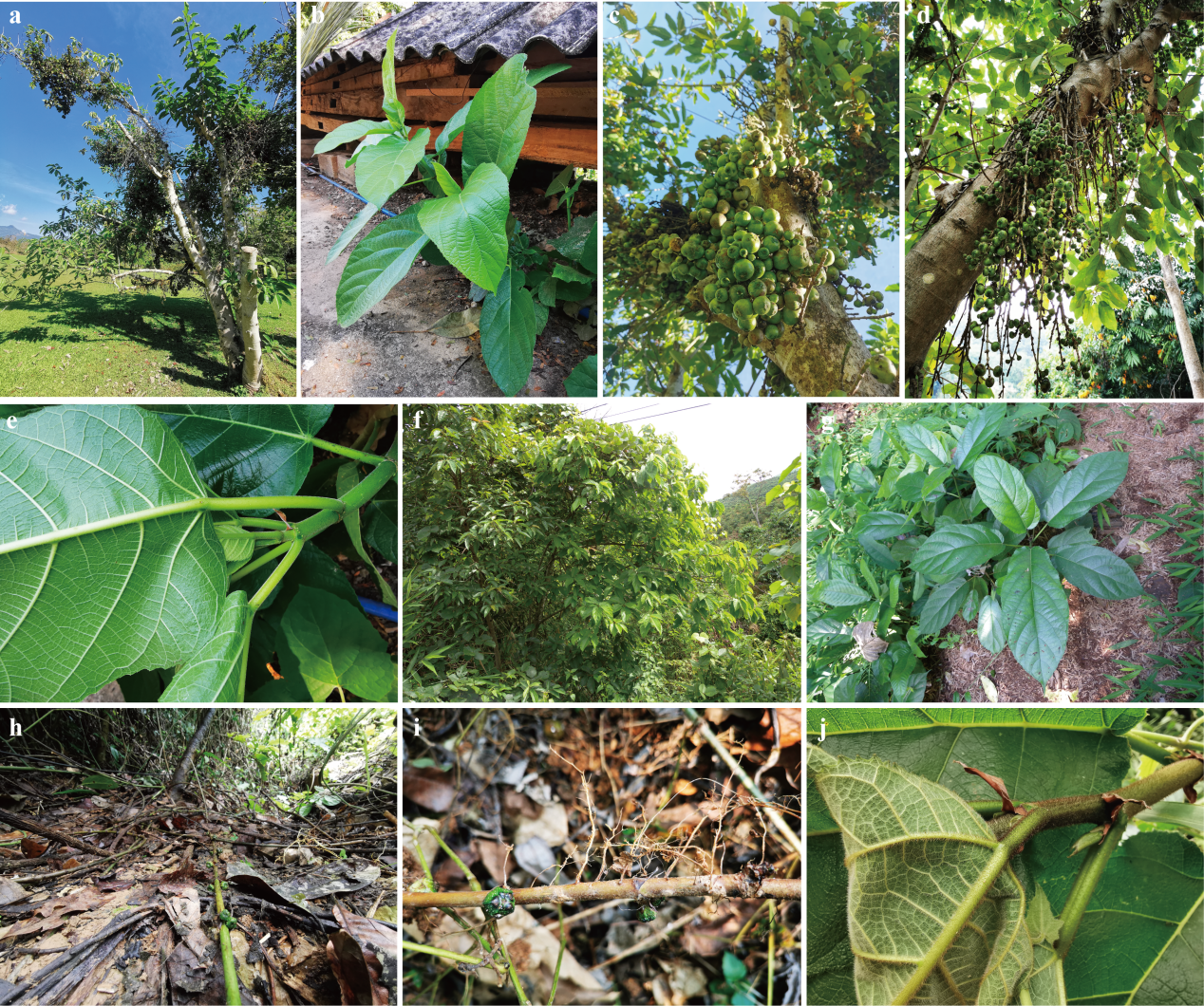


**Figure S1** Study species. *Ficus hispida*: (a) adult individual, (b) young individual, (c-d) cauliflorous or ramiflorous figs, (e) whitish puberulous; *Ficus heterostyla*: (f) adult individual, (g) young individual, (h-i) stoloniflory figs, (j) whitish puberulous, intermixed with longer patent dark brown to purplish hairs.


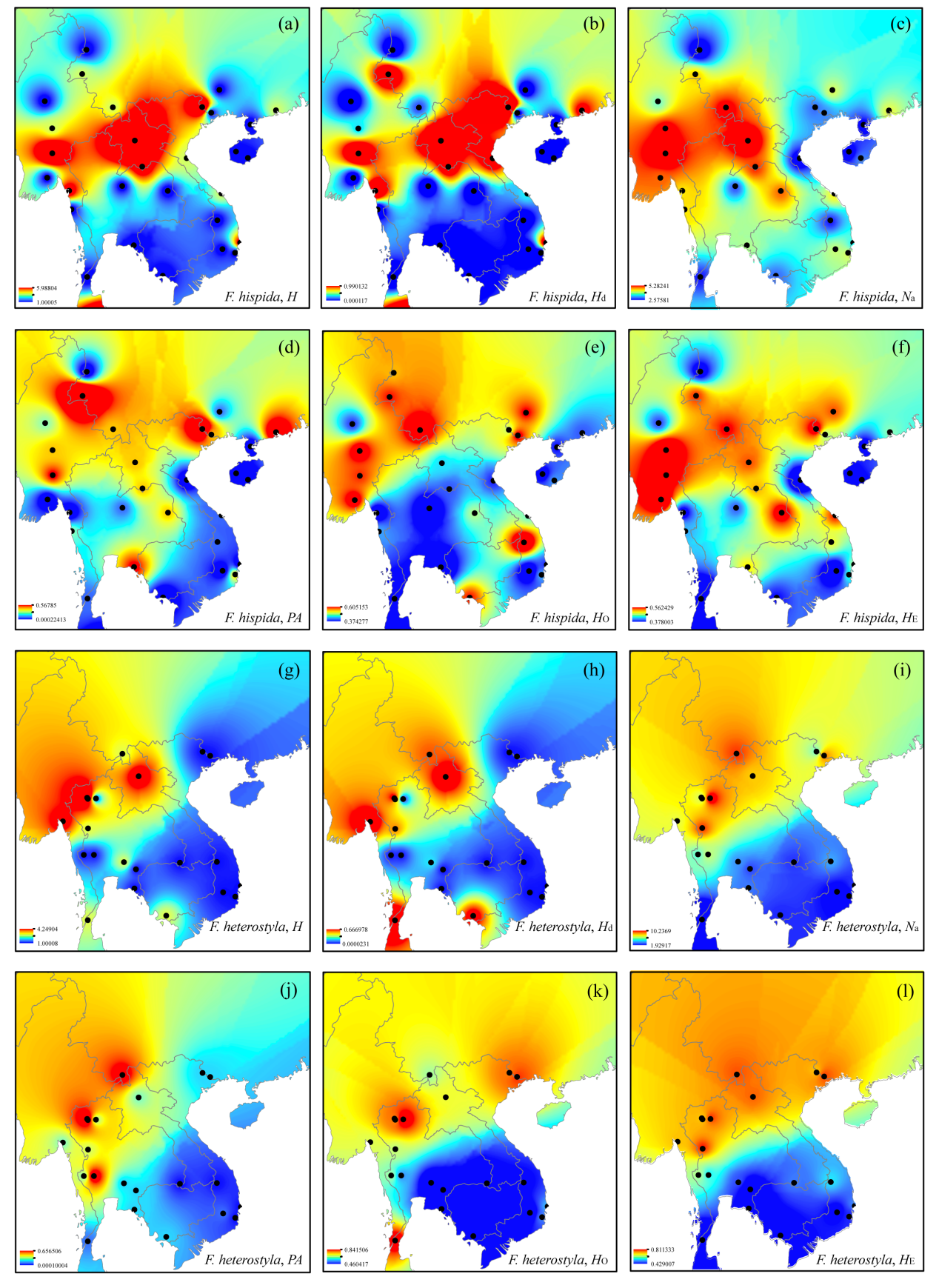


**Figure S2** Genetic diversity maps of the sampled *F. hispida* and *F. heterostyla* populations (black dots) in Indo-Burma: IDW interpolation of the number of haplotypes (a), haplotype diversity (b), number of alleles (c), private alleles per locus (d), observed (e) and expected heterozygosity (f) for *F. hispida*; IDW interpolation of the number of haplotypes (g), haplotype diversity (h), number of alleles (i), private alleles per locus (j), observed (k) and expected heterozygosity (l) for *F. heterostyla*.


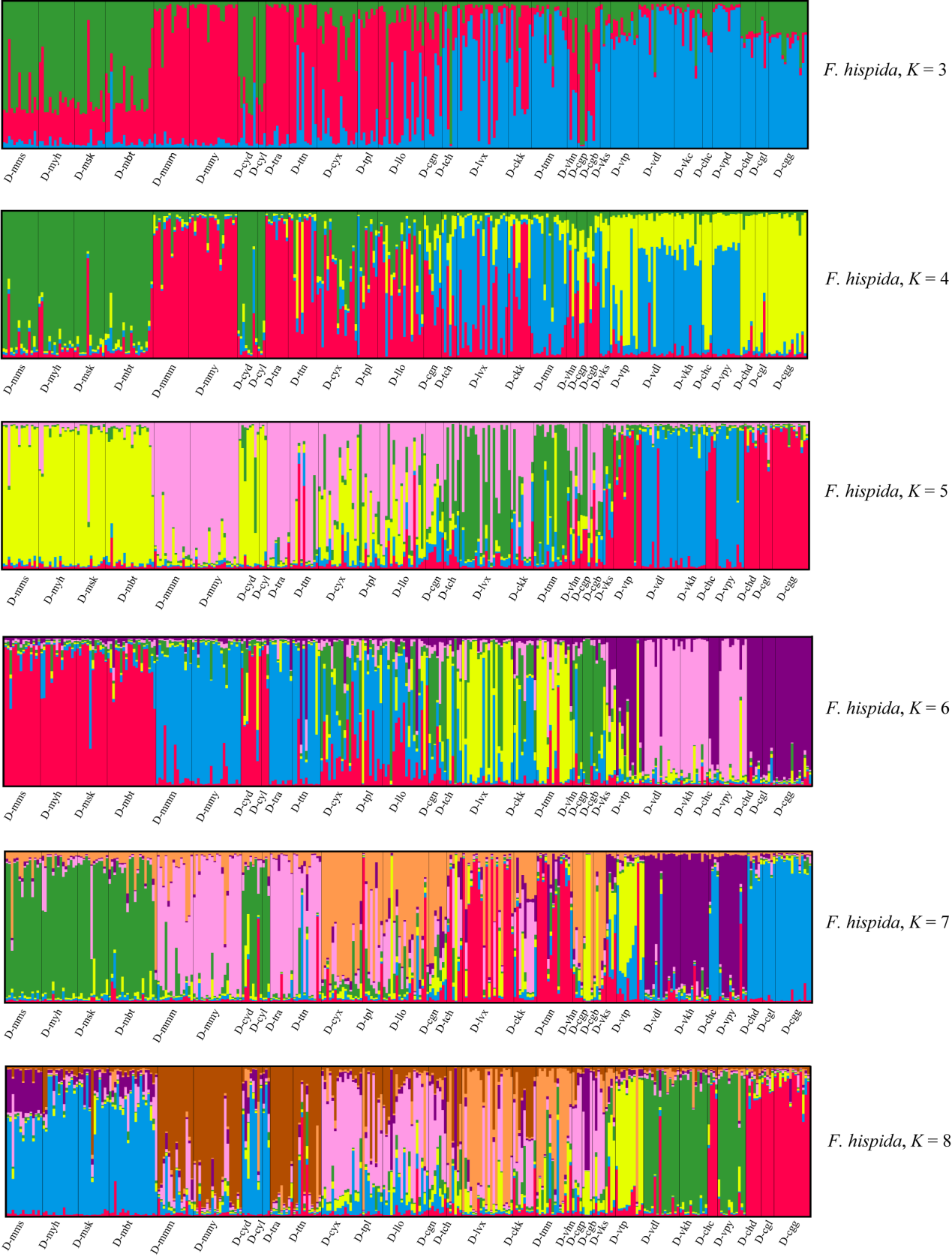


**Figure S3** The bar plots of the membership probabilities of *F. hispida* individuals to the different clusters from the STRUCTURE analysis at *K* = 3 to 8 based on 14 nSSRs.


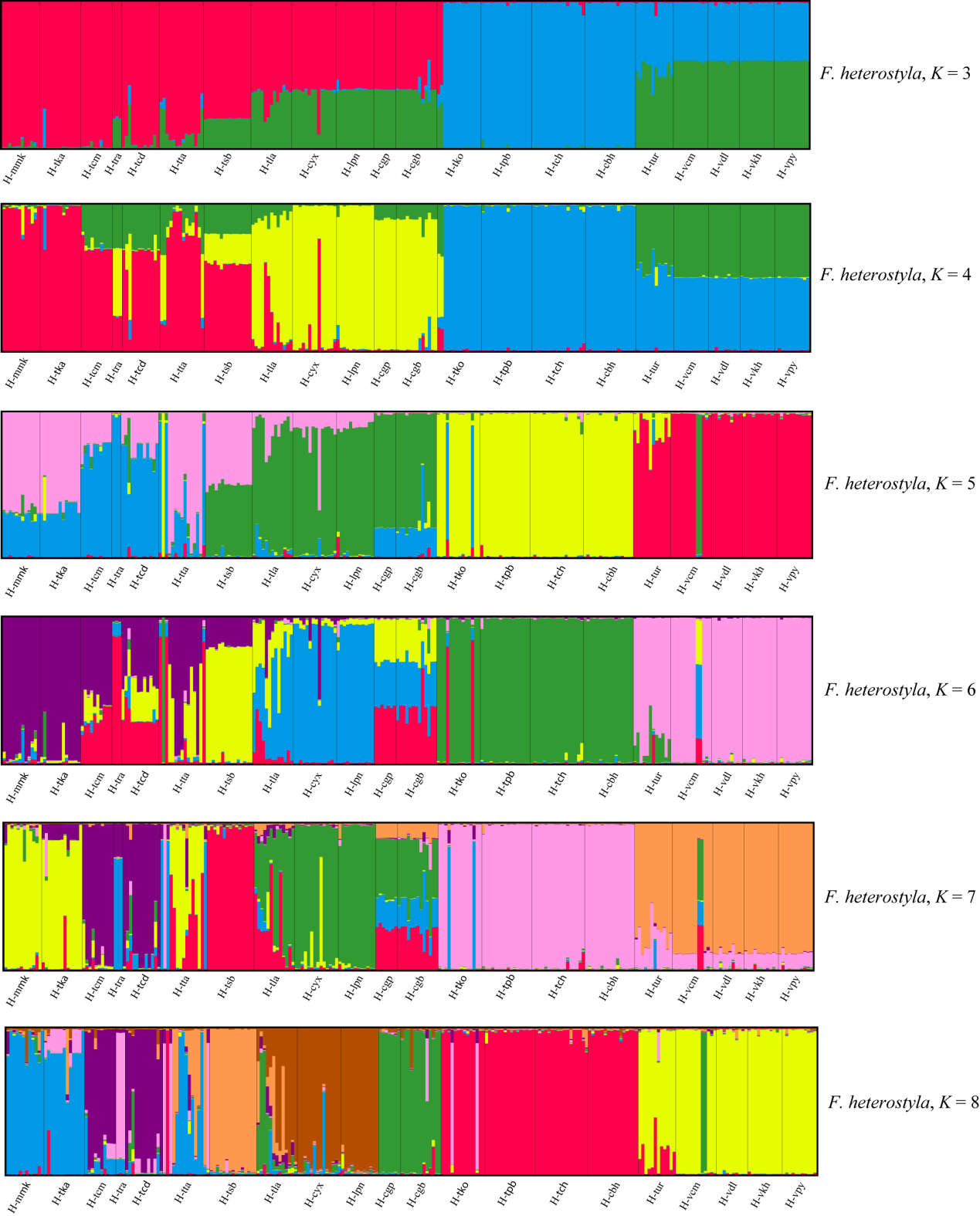


**Figure S4** The bar plots of the membership probabilities of *F. heterostyla* individuals to the different clusters from the STRUCTURE analysis at *K* = 3 to 8 based on 14 nSSRs.


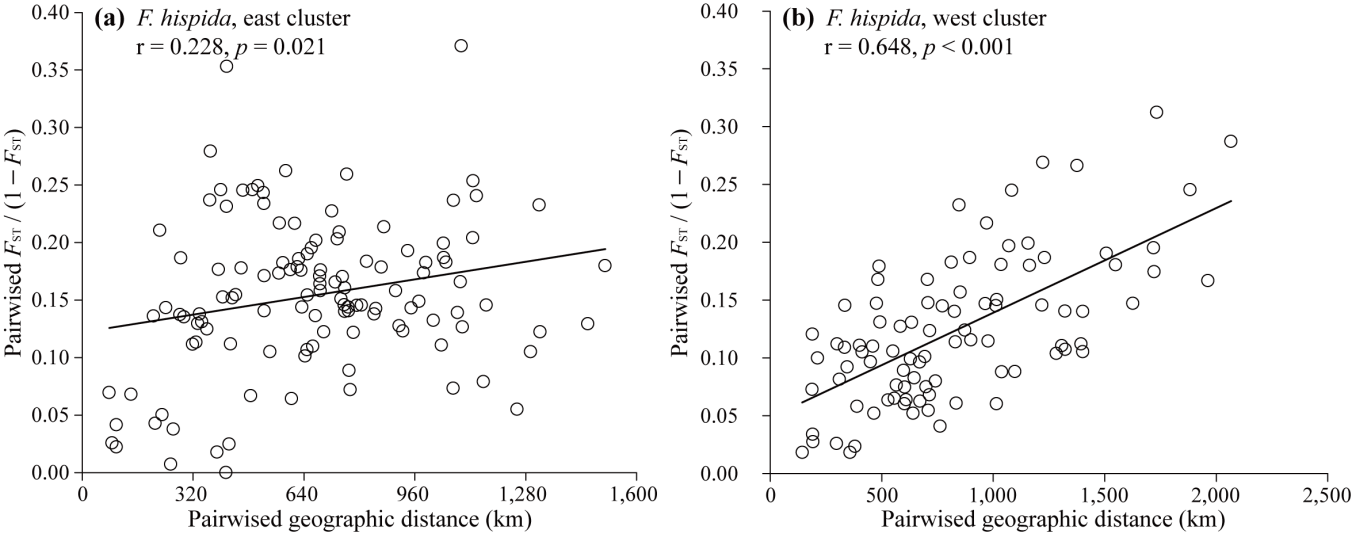


**Figure S5** The regression of paired *F*_ST_/(1-*F*_ST_) vs the geographic distance was significant for nuclear microsatellites data in both east (a) and west (b) *F. hispida* clusters.


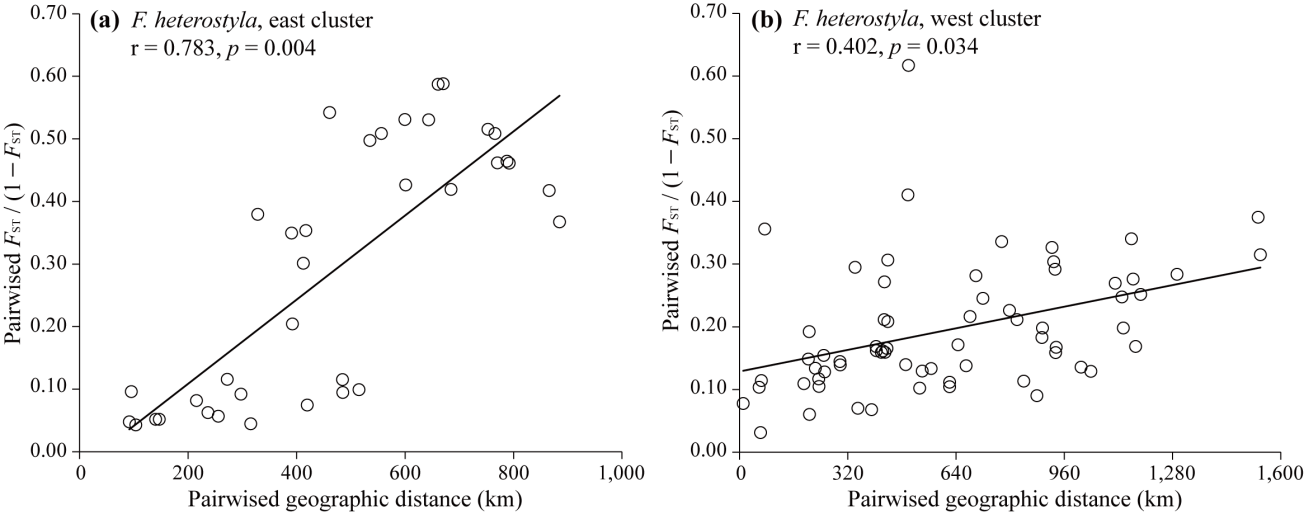


**Figure S6** The regression of paired *F*_ST_/(1-*F*_ST_) vs the geographic distance was significant for nuclear microsatellites data in both east (a) and west (b) *F. heterostyla* clusters.


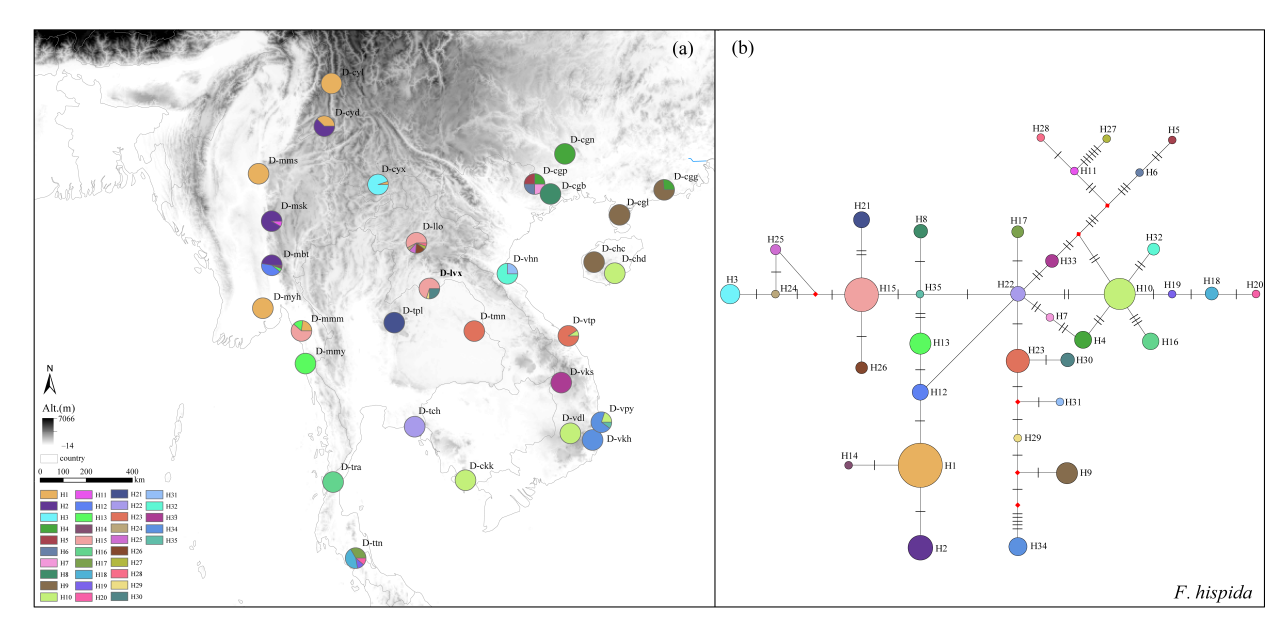


**Figure S7** Maps with distributions of cpDNA haplotypes of *F. hispida* (a) and the median-joining network cpDNA haplotypes of *F. hispida* (b). The size of each circle in (b) represents the frequency of each haplotype. Each bar on the lines connecting two haplotypes represents one mutational step and the small red diamond nodes on the lines represent inferred missing haplotypes.


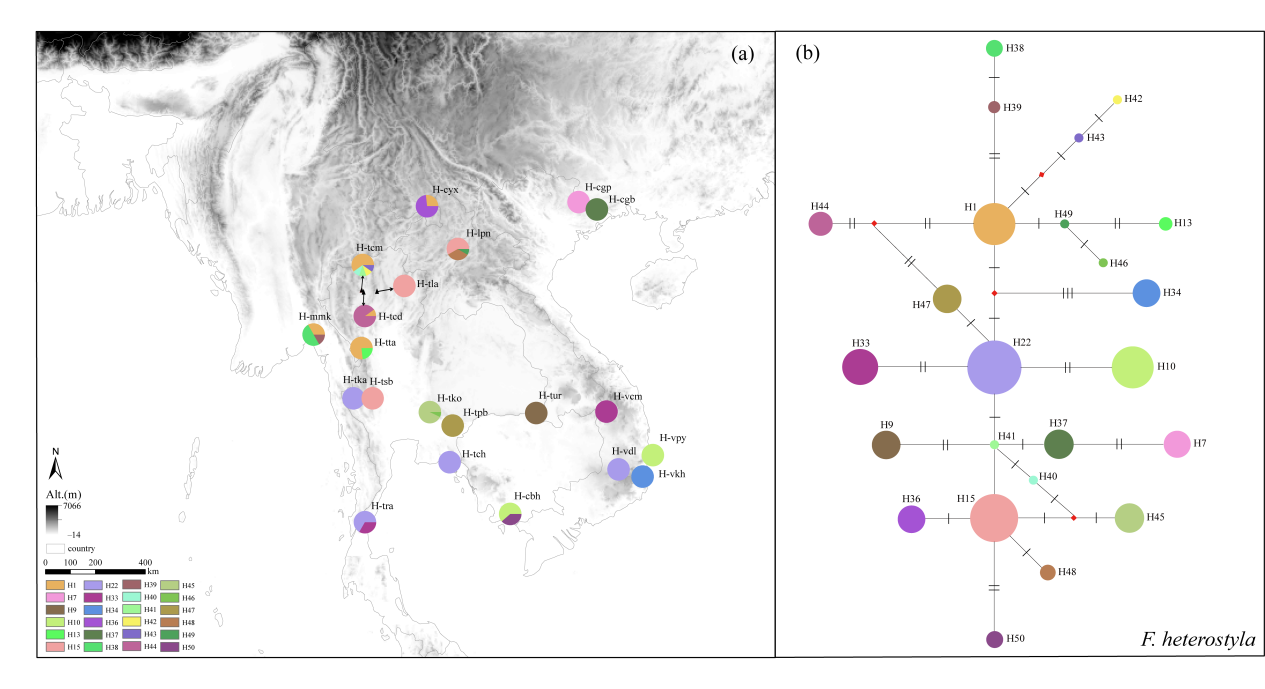


**Figure S8** Maps with distributions of cpDNA haplotypes of *F. heterostyla* (a) and the median-joining network cpDNA haplotypes of *F. heterostyla* (b). The size of each circle in (b) represents the frequency of each haplotype. Each bar on the lines connecting two haplotypes represents one mutational step and the small red diamond nodes on the lines represent inferred missing haplotypes.


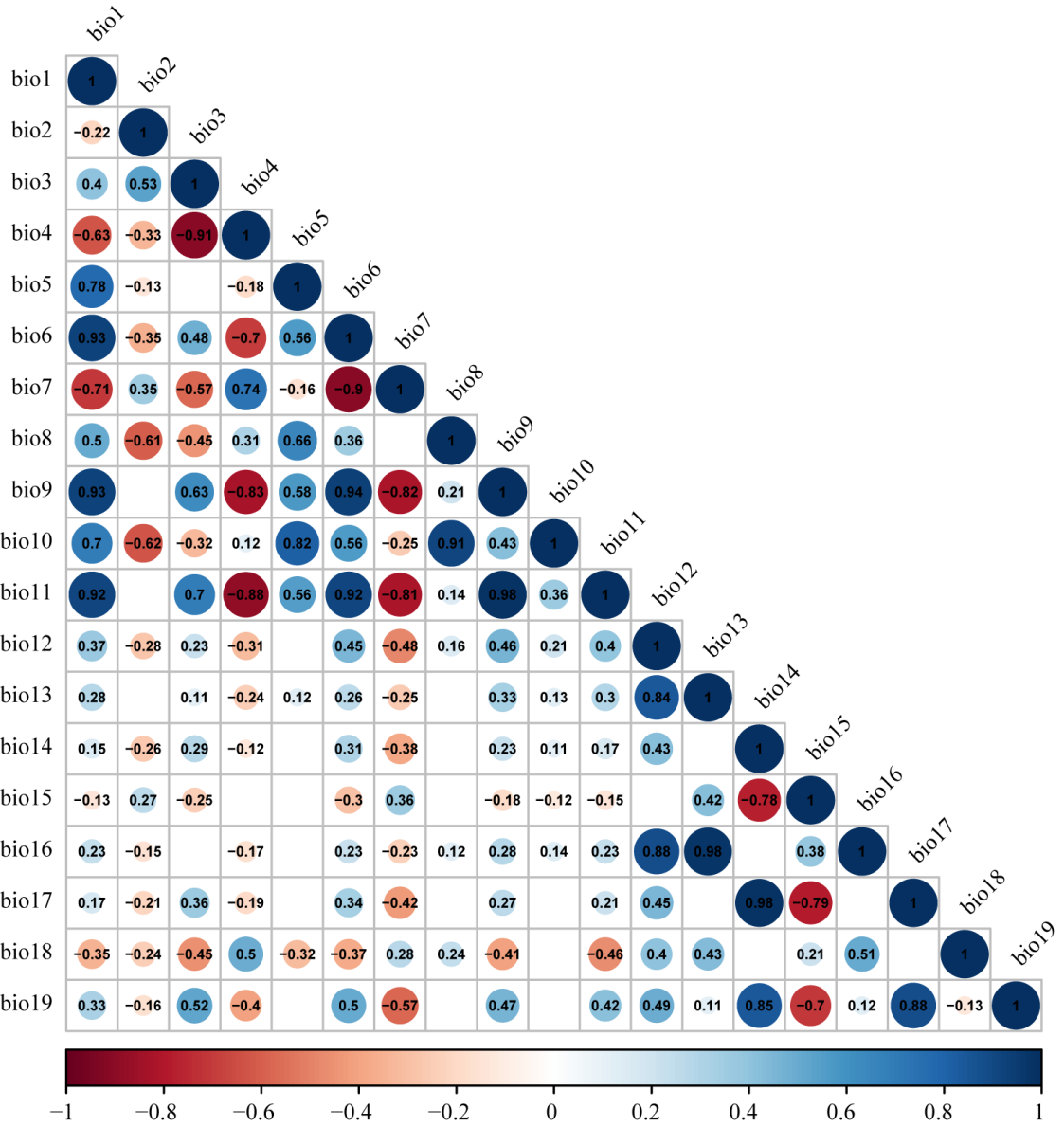


**Figure S9** Pearson correlation matrix among the nineteen biologic climatic variables defined by WorldClim. Significant correlations (p < 0.01) are indicated and colored. The sizes of colored pies and the change in color depth are proportional to the absolute values of the correlation coefficient.


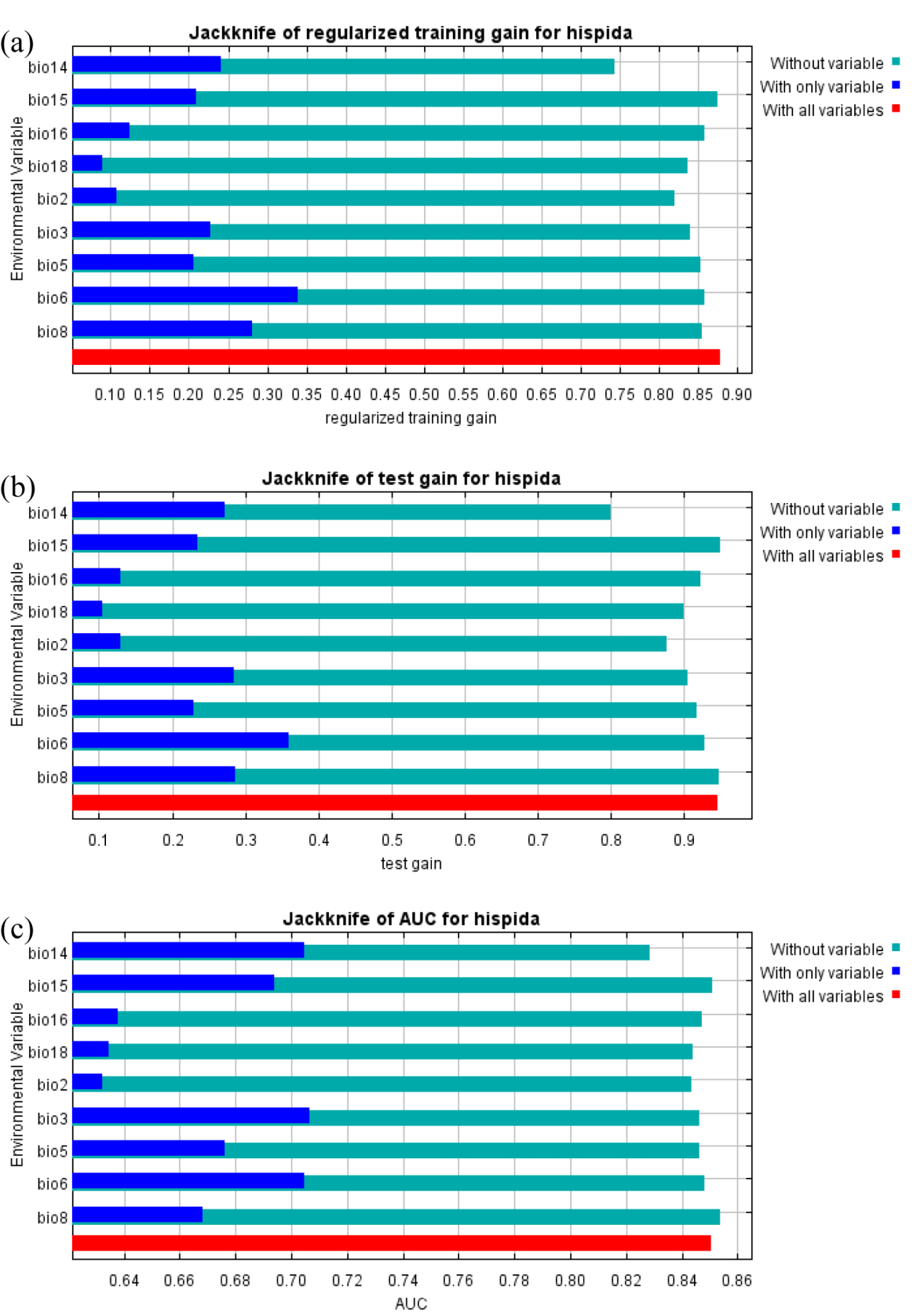


**Figure S10** Jackknife test for regularized training gain (a), test gain (b) and AUC (c) of each environmental variable importance for *F. hispida* in present period; blue bar indicates a model created using only one variables, while a model with the remaining variables is indicated with light blue bar. The red bar indicates the model created using all variables.
