# Supplementary material for: East‒West genetic differentiation across the Indo-Burma hotspot: evidence from two closely related dioecious figs
Source: BMC Plant Biol. 2023 Jun 16;23:321. doi: 10.1186/s12870-023-04324-6 (PMC10273766; doi:10.1186/s12870-023-04324-6)
Supplement: Supplementary file 2 — Supplementary Material 2 [file 12870_2023_4324_MOESM2_ESM.docx]

| **Species** | ***K*** | ***F*_CT_** | **Population grouping** |
| --- | --- | --- | --- |
| *F. hispida* | 2 | 0.13490 | (D-ckk, D-cgl, D-cgb, D-cgp, D-cgg, D-chc, D-chd, D-tmn, D-tch, D-lvx, D-vtp, D-vks, D-vkh, D-vhn, D-vpy, D-ttn, D-cgn, D-llo, D-mmy, D-mbt, D-mmm, D-mms, D-myh, D-tpl, D-cyd, D-cyx, D-vdl, D-tra) (D-msk, D-cyl) |
|  | 3 | 0.11132 | (D-ckk, D-cgl, D-cgb, D-cgp, D-cgg, D-chc, D-chd, D-tmn, D-tch, D-lvx, D-vtp, D-vks, D-vkh, D-vhn, D-vpy, D-ttn, D-cgn, D-llo, D-mmy, D-mbt, D-mmm, D-mms, D-myh, D-tpl, D-cyd, D-vdl, D-cyx) (D-msk, D-cyl, D-tra) (D-vdl) |
|  | 4 | 0.11712 | (D-ckk, D-cgl, D-cgb, D-cgp, D-cgg, D-chc, D-chd, D-tmn, D-tch, D-lvx, D-vtp, D-vks, D-vkh, D-vhn, D-vpy, D-ttn, D-cgn, D-llo, D-mmy, D-mbt, D-mmm, D-mms, D-tpl, D-cyd, D-vdl, D-cyx) (D-msk, D-myh, D-cyl) (D-vdl) (D-tra) |
|  | 5 | 0.11795 | (D-ckk, D-cgb, D-cgp, D-cgg, D-chc, D-chd, D-tmn, D-tch, D-lvx, D-vtp, D-vks, D-vkh, D-vhn, D-vpy, D-ttn, D-cgn, D-llo, D-mmy, D-mbt, D-mmm, D-mms, D-tpl, D-cyd, D-vdl, D-cyx) (D-msk, D-myh, D-cyl) (D-vdl) (D-tra) (D-cgl) |
|  | 6 | 0.11763 | (D-ckk, D-cgb, D-cgp, D-cgg, D-chc, D-chd, D-tmn, D-tch, D-lvx, D-vtp, D-vks, D-vkh, D-vhn, D-vpy, D-ttn, D-cgn, D-llo, D-mmy, D-mbt, D-mmm, D-mms, D-tpl, D-cyd, D-vdl, D-cyx) (D-msk, D-myh) (D-cyl) (D-vdl) (D-tra) (D-cgl) |
|  | 7 | 0.11578 | (D-ckk, D-cgb, D-cgg, D-chc, D-chd, D-tmn, D-tch, D-lvx, D-vtp, D-vks, D-vkh, D-vhn, D-vpy, D-ttn, D-cgn, D-llo, D-mmy, D-mbt, D-mmm, D-mms, D-tpl, D-cyd, D-vdl, D-cyx) (D-msk, D-myh) (D-cyl) (D-vdl) (D-tra) (D-cgl) (D-cgp) |
|  | 8 | 0.11365 | (D-cgb, D-cgg, D-chc, D-chd, D-tmn, D-tch, D-lvx, D-vtp, D-vks, D-vkh, D-vhn, D-vpy, D-ttn, D-cgn, D-llo, D-mmy, D-mbt, D-mmm, D-mms, D-tpl, D-cyd, D-vdl, D-cyx) (D-msk, D-myh) (D-cyl) (D-vdl) (D-tra) (D-cgl) (D-cgp) (D-ckk) |
|  | 9 | 0.11154 | (D-cgb, D-cgg, D-chc, D-chd, D-tmn, D-tch, D-lvx, D-vks, D-vkh, D-vhn, D-vpy, D-ttn, D-cgn, D-llo, D-mmy, D-mbt, D-mmm, D-mms, D-tpl, D-cyd, D-vdl, D-cyx) (D-msk, D-myh) (D-cyl) (D-vdl) (D-tra) (D-cgl) (D-cgp) (D-ckk) (D-vtp) |
|  | 10 | 0.10874 | (D-cgb, D-cgg, D-chc, D-tmn, D-tch, D-lvx, D-vks, D-vkh, D-vhn, D-vpy, D-ttn, D-cgn, D-llo, D-mmy, D-mbt, D-mmm, D-mms, D-tpl, D-cyd, D-vdl, D-cyx) (D-msk, D-myh) (D-cyl) (D-vdl) (D-tra) (D-cgl) (D-cgp) (D-ckk) (D-vtp) (D-chd) |
| *F. heterostyla* | 2 | 0.22973 | (H-lpn, H-cyx, H-tla, H-tta, H-tsb, H-cgb, H-cgp, H-tcd, H-tcm, H-mmk, H-tka, H-tra) (H-tko, H-tch, H-tpb, H-cbh, H-tur, H-vpy, H-vdl, H-vkh, H-vcm) |
|  | 3 | 0.29841 | (H-lpn, H-cyx, H-tla, H-tta, H-tsb, H-cgb, H-cgp, H-tcd, H-tcm, H-mmk, H-tka, H-tra) (H-tko, H-tch, H-tpb, H-cbh) (H-tur, H-vpy, H-vdl, H-vkh, H-vcm) |
|  | 4 | 0.30621 | (H-lpn, H-cyx, H-tla, H-tta, H-tsb, H-cgb, H-cgp, H-tcd, H-tcm) (H-mmk, H-tka, H-tra) (H-tko, H-tch, H-tpb, H-cbh) (H-tur, H-vpy, H-vdl, H-vkh, H-vcm) |
|  | 5 | 0.31110 | (H-lpn, H-cyx, H-tla, H-tta, H-tsb, H-cgb, H-cgp) (H-tcm, H-tcd) (H-mmk, H-tka, H-tra) (H-tko, H-tch, H-tpb, H-cbh) (H-tur, H-vpy, H-vdl, H-vkh, H-vcm) |
|  | 6 | 0.31582 | (H-lpn, H-cyx, H-tla, H-tta, H-tsb) (H-cgb, H-cgp) (H-tcm, H-tcd) (H-mmk, H-tka, H-tra) (H-tko, H-tch, H-tpb, H-cbh) (H-tur, H-vpy, H-vdl, H-vkh, H-vcm) |
|  | 7 | 0.32068 | (H-lpn, H-cyx, H-tla, H-tta) (H-tsb) (H-cgb, H-cgp) (H-tcm, H-tcd) (H-mmk, H-tka, H-tra) (H-tko, H-tch, H-tpb, H-cbh) (H-tur, H-vpy, H-vdl, H-vkh, H-vcm) |
|  | 8 | 0.32826 | (H-lpn, H-cyx) (H-tla, H-tta) (H-tsb) (H-cgb, H-cgp) (H-tcm, H-tcd) (H-mmk, H-tka, H-tra) (H-tko, H-tch, H-tpb, H-cbh) (H-tur, H-vpy, H-vdl, H-vkh, H-vcm) |
|  | 9 | 0.33298 | (H-lpn, H-cyx) (H-tla, H-tta) (H-tsb) (H-cgb, H-cgp) (H-tcm, H-tcd) (H-mmk, H-tka, H-tra) (H-tko, H-tch, H-tpb) (H-cbh) (H-tur, H-vpy, H-vdl, H-vkh, H-vcm) |
|  | 10 | 0.33412 | (H-lpn) (H-cyx) (H-tla, H-tta) (H-tsb) (H-cgb, H-cgp) (H-tcm, H-tcd) (H-mmk, H-tka) (H-tra) (H-tko, H-tch, H-tpb, H-cbh) (H-tur, H-vpy, H-vdl, H-vkh, H-vcm) |

**Table S1** *F*_CT_ values for different numbers of population groups (*K*) inferred by the SAMOVA algorithm using the microsatellite dataset

**Table S2** Median estimation of posterior distributions for each scenario based on Approximate Bayesian Computation (ABC)

| Species | Parameter | Scenario 1 | Scenario 2 | Scenario 3 | Scenario 4 |
| --- | --- | --- | --- | --- | --- |
| *F. hispida* | Posterior probability | 0.6363 | 0.1826 | 0.1600 | 0.0211 |
|  | t1 | – | 542 | – | 438 |
|  | t_2_ | – | – | 674 | 615 |
|  | t_3_ | 1250 | 1060 | 1010 | 764 |
|  | *N*_1_ | 7040 | 8240 | 6910 | 7810 |
|  | *N*_1a_ | – | 3840 | – | 3780 |
|  | *N*_2_ | 8810 | 9080 | 9050 | 8880 |
|  | *N*_2a_ | – | – | 4450 | 4310 |
|  | *N*_3_ | 1050 | 1060 | 1180 | 980 |
| *F. heterostyla* | Posterior probability | 0.1837 | 0.4564 | 0.0913 | 0.2686 |
|  | t_1_ | – | 1380 | – | 1340 |
|  | t_2_ | – | – | 3310 | 4030 |
|  | t_3_ | 4530 | 4910 | 5650 | 6080 |
|  | *N*_1_ | 2250 | 5250 | 2140 | 5200 |
|  | *N*_1a_ | – | 1350 | – | 1780 |
|  | *N*_2_ | 9810 | 9780 | 9790 | 9990 |
|  | *N*_2a_ | – | – | 7750 | 6260 |
|  | *N*_3_ | 1260 | 1430 | 750 | 688 |

*N*_1_, *N*_1a_, t_1_: The effective population size of east cluster at present and at t_1_, respectively; *N*_2,_ *N*_2a_, t_2_: The effective population size of west cluster at present and at t_2_, respectively; *N*_3_, t_3_: The effective population size of ancestral populations at t_3_. The time (t) parameters were estimated in generations.

**Table S3** The nine bioclimatic variables with Pearson correlation coefficients of |r| ≤ 0.8 and their percent contribution and permutation importance in predicting the distribution of *F. hispida*

| Bioclim codes | Bioclimatic variables | Contribution (%) | Permutation |
| --- | --- | --- | --- |
| Bio2 | Mean Diurnal Range (Mean of monthly (max temp - min temp)) | 7.4 | 5.7 |
| Bio3 | Isothermality (BIO2/BIO7)  (×100) | 21.5 | 16.7 |
| Bio5 | Maximum Temperature of Warmest Month | 2.9 | 10.7 |
| Bio6 | Minimum Temperature of Coldest Month | 23.2 | 7 |
| Bio8 | Mean Temperature of Wettest Quarter | 12.2 | 21.4 |
| Bio14 | Precipitation of Driest Month | 23.8 | 25.7 |
| Bio15 | Precipitation Seasonality (Coefficient of Variation) | 2.4 | 1.8 |
| Bio16 | Precipitation of Wettest Quarter | 2.1 | 3.2 |
| Bio18 | Precipitation of Warmest Quarter | 4.6 | 7.8 |

**Table S4** GenBank accessions of the 50 hyplotypes isolated from *F. hispida* and *F. heterostyla*, and outgroup taxa.

| Taxa | | haplotypes | GenBank accessions | |
| --- | --- | --- | --- | --- |
|  |  |  | psbA-trnH | trnS-trnG |
| tribe Ficeae | *Ficus hispida* + *F. heterostyla* | H1–H50 | OQ296142–OQ296191 | OQ296192–OQ296241 |
| tribe Castilleae | *Castilla elastica* |  | KU855632 | KU855854 |
|  | *Poulsenia armata* |  | MK914203 | na |
|  | *Sparattosyce dioica* |  | KU855665 | KU855862 |
